# Maintenance of tRNA and elongation factors supports T3SS proteins translational elongations in pathogenic bacteria during nutrient starvation

**DOI:** 10.1186/s13578-022-00884-6

**Published:** 2022-09-05

**Authors:** Yue Sun, Xiaolong Shao, Yingchao Zhang, Liangliang Han, Jiadai Huang, Yingpeng Xie, Jingui Liu, Xin Deng

**Affiliations:** 1 Department of Biomedical Sciences, City University of Hong Kong, Kowloon Tong, Hong Kong SAR, China; 2 College of Plant Protection, Laboratory of Plant Immunity, Key Laboratory of Integrated Management of Crop Diseases and Pests, Nanjing Agricultural University, No. 1 Weigang, Nanjing, 210095 Jiangsu China; 3 Shenzhen Research Institute, City University of Hong Kong, Shenzhen, 518057 China

**Keywords:** Pathogenic bacteria, T3SS, Translational elongation rate, Nutrient-limiting conditions, tRNA, Elongation factor

## Abstract

**Background:**

Sufficient nutrition contributes to rapid translational elongation and protein synthesis in eukaryotic cells and prokaryotic bacteria. Fast synthesis and accumulation of type III secretion system (T3SS) proteins conduce to the invasion of pathogenic bacteria into the host cells. However, the translational elongation patterns of T3SS proteins in pathogenic bacteria under T3SS-inducing conditions remain unclear. Here, we report a mechanism of translational elongation of T3SS regulators, effectors and structural protein in four model pathogenic bacteria (*Pseudomonas syringae*, *Pseudomonas aeruginosa*, *Xanthomonas oryzae* and *Ralstonia solanacearum*) and a clinical isolate (*Pseudomonas aeruginosa* UCBPP-PA14) under nutrient-limiting conditions. We proposed a luminescence reporter system to quantitatively determine the translational elongation rates (ERs) of T3SS regulators, effectors and structural protein under different nutrient-limiting conditions and culture durations.

**Results:**

The translational ERs of T3SS regulators, effectors and structural protein in these pathogenic bacteria were negatively regulated by the nutrient concentration and culture duration. The translational ERs in 0.5× T3SS-inducing medium were the highest of all tested media. In 1× T3SS-inducing medium, the translational ERs were highest at 0 min and then rapidly decreased. The translational ERs of T3SS regulators, effectors and structural protein were inhibited by tRNA degradation and by reduced levels of elongation factors (EFs).

**Conclusions:**

Rapid translational ER and synthesis of T3SS protein need adequate tRNAs and EFs in nutrient-limiting conditions. Numeric presentation of T3SS translation visually indicates the invasion of bacteria and provides new insights into T3SS expression that can be applied to other pathogenic bacteria.

**Supplementary Information:**

The online version contains supplementary material available at 10.1186/s13578-022-00884-6.

## Introduction

Bacterial growth primarily depends on protein synthesis catalyzed by the ribosome. Protein accounts for half of the bacterial biomass, and its synthesis requires more than 60% of the total cellular energy [1, 2]. Bacterial growth and survival can be suppressed by many environmental stressors, such as nutrient deficiency, oxidative stress, low temperatures and high osmotic pressure [3–6]. The bacterial growth rate depends on many factors: the ribosome concentration, the ribosome translational elongation rate (ER) and other factors, such as tRNAs, and ribosome-affiliated factors [1, 7]. Translational elongation comprises four basic steps. In the first step, mRNA codons recognise and regulate tRNAs. In the second step, tRNA is transferred to the ribosome by the elongation factor (EF) Tu. In the third step, the polypeptide chain is assembled with amino acids, catalyzed by tRNA. Finally, the movement of deacylated tRNA results in ribosome translocation [8]. During the translation, ribosomes move at the uneven rates along the mRNAs [9]. The dynamic of translational elongation contributes to the protein folding [10, 11]. Translational elongation is essential to the physiological regulation as the elongation stage consumes great mass of energy for protein synthesis [12]. The unstable translational elongation process can lead to diseases, such as cancers [13, 14]. In bacteria, the fluctuation of translational ER changes the speed of ribosome movement that affects the protein synthesis [15, 16]. Under nutrient-limiting conditions, the protein synthesis rate and bacterial growth are severely inhibited [17, 18].

The activity of the bacterial type III secretion system (T3SS) is regulated by environmental nutrition conditions or the host environment and enables bacterial pathogens to invade host cells [19, 20]. In Gram-negative bacteria such as *Pseudomonas syringae*, *Pseudomonas aeruginosa*, *Xanthomonas oryzae* and *Ralstonia solanacearum*, the highly conserved T3SS is the key factor allowing the establishment of infection. The T3SS needle-like machinery delivers effector proteins into host cells to suppress the host immune system [21, 22]. Bacterial infection of plants results in substantial crop yield losses [23–25], while in humans, bacteria cause several types of infectious diseases [26, 27]. In general, T3SS is suppressed under nutrient-rich conditions but induced under nutrient-poor conditions, such as intercellular spaces [28–30].

Many T3SS regulators have been identified in recent decades [31–34]. In the case of the opportunistic human pathogen *P. aeruginosa*, the T3SS genes are induced by contact with host cells or by low-calcium conditions, which can be achieved by adding 5 mM ethylene glycol tetraacetic acid (EGTA) to LB [35, 36]. The T3SS genes are directly regulated by ExsA, which autoregulates its expression by binding to the promoters and activating their transcription [19]. ExsA transcriptional activation can be inhibited by the anti-activators ExsD and PtrA [37, 38]. PopN is a repressor of T3SS that is the first gene of the operon *popN*-*pcr1*-*pcr2*-*pcr3*-*pcr4*-*pcrD*-*pcrR*-*pcrG*-*pcrV*-*pcrH*-*popB*-*popD*. PopN contributes to detecting the intracellular calcium levels [39]. PopN interacts with Pcr1 and suppresses the T3SS expression in *P. aeruginosa* [39, 40].

In plant pathogens, T3SS is encoded by the clustered hypersensitive response (*hrc*) and pathogenicity (*hrp*) genes, which are divided into two groups. Group I includes the *hrp* clusters of *P. syringae*, while group II contains those of *X. oryzae* and *R. solanacearum* [41]. Most T3SS genes are directly regulated by the alternative sigma factor HrpL, which binds to the *hrp* box in the T3SS gene promoter region [42, 43]. *hrpL* transcription is activated through the regulation of another alternative sigma factor, RpoN [44]. *hrpL* transcription is also autoregulated through the bi-directional promoter region between *hrpL* and *hrpJ* [45]. Heterodimerised HrpRS binds to the *hrpL* promoter and activates *hrpL* expression [46]. HrpS can also directly activate the transcription of T3SS genes independent of HrpR [47]. The *hrpRS* gene operon is positively regulated by HrpA, a component of the T3SS pilus, and the extra-cytoplasmic function sigma factor AlgU [48, 49]. HrpRS is modulated by at least six two-component systems (TCSs): RhpRS, CvsRS, GacAS, AauRS, CbrAB2 and EnvZ-OmpR [32, 50–53]. In addition, a variety of regulators positively or negatively modulate T3SS genes. The protease Lon suppresses T3SS by degrading HrpR and other effectors secreted by T3SS [54, 55]. The HrpGVFJ regulatory pathway controls the formation of HrpRS heterodimer at the post-transcriptional level [56–59]. Many transcription factors, such as PilR, TrpI and GntR, act as virulence regulators by influencing T3SS [60]. HrpA2 is one of the major T3SS structural proteins and activates the hypersensitive reaction and pathogenesis in the plants, which shows a significant role in the *P. syringae* pv. *tomato* DC3000 [61]. *hopG1* gene encodes a T3SS effector protein that localizes to the mitochondria. HopG1 promotes the growth of pathogens and inhibits the plant defense responses [62, 63]. T3SS effector HopX1 is a cysteine protease. In *P. syringae* pv. *tabaci*, HopX1 facilitates the susceptibility through directly degrading jasmonate pathway repressors [64, 65].

In *P. syringae*, RhpRS is a master regulatory TCS of T3SS [32, 66]. The *rhpS* and *rhpR* genes are located in the same operon; *rhpS* encodes a histidine kinase and *rhpR* encodes a cognate response regulator [66]. Phosphorylated RhpR directly binds to the *rhpRS* promoter and activates *rhpRS* expression to regulate a group of virulence-related phenotypes [34]. As an important negative response regulator of *hrpRS*, phosphorylated RhpR binds to the *hrpRS* promoter to suppress the *hrpRS*-*hrpL*-*hrp* gene cascade under different environmental conditions [31, 32, 34, 67]. RhpS senses changes in environmental conditions and dephosphorylates phosphorylated RhpR, thereby suppressing *rhpRS* expression [31, 66]. We recently found that RhpS also senses polyphenols to inhibit the expression of T3SS and *P. syringae* virulence [68].

In *X. oryzae*, Hpa1 is an *hrp*-dependent secreted protein that is regulated by two genes, *hrpG* and *hrpX* [69, 70]. HrpX directly activates the expression of *hrp* genes by recognizing the consensus sequence in its promoters [71]. In *R. solanacearum*, HrpG acts as a TCS response regulator that activates the production of plant hormones and regulates the expression of virulence genes involved in bacterial protection responses and plant cell wall degradation [72]. In addition, HrpG positively regulates the transcription of HrpB, which is called HrpX in *X. oryzae* [73].

Although many T3SS regulators have been identified, the translational elongation capacity of T3SS under nutrient-limiting conditions remains uncertain. However, this process plays a considerable role in T3SS protein regulation and bacterial infection. Here, we identified a mechanism in which the T3SS translational ER in four model bacteria is negatively regulated by the nutrient conditions and culture durations. The ER under nutrient-limiting conditions was suppressed by tRNA degradation and reduced levels of EFs. This unified mechanism of T3SS protein translational elongation under nutrient-limiting conditions indicates that such conditions would hinder bacterial infection.

## Results

### Translation patterns of T3SS regulators, effectors and structural protein of pathogenic bacteria

The translational ER is an accurate indicator for evaluating protein synthesis [4]. Elongation is required for synthesizing proteins under nutrient-limiting conditions in *Escherichia coli*, even though it occurs at a slow rate [74]. However, the translational ERs of T3SS proteins and the associated inhibitory factors in pathogenic bacteria remain unclear. Thus, we evaluated the ERs of T3SS regulators, effectors and structural protein in *P. syringae* (PS), *P. aeruginosa* (PA), *X. oryzae* (XO) and *R. solanacearum* (RS). Here, we constructed two kinds of reporters. One included the corresponding promoter and a promoter-less luciferase (*lux*) in the plasmid, which was the transcriptional fusion reporter to calculate the transcription time (*T*_*initiation*_). The other reporter included the corresponding promoter, the coding sequence (CDS) and a promoter-less luciferase (*lux*) in the plasmid, which was the reporter for both transcription and translation to calculate the time for both processes (*T*_*test*_). The translational elongation time (*T*_*first*_) of target protein was calculated by *T*_*test*_ minus *T*_*initiation*_. The translational ERs of the tested proteins equaled protein length (*L*) divided by *T*_*first*_ [4, 75].

**Fig. 1:**
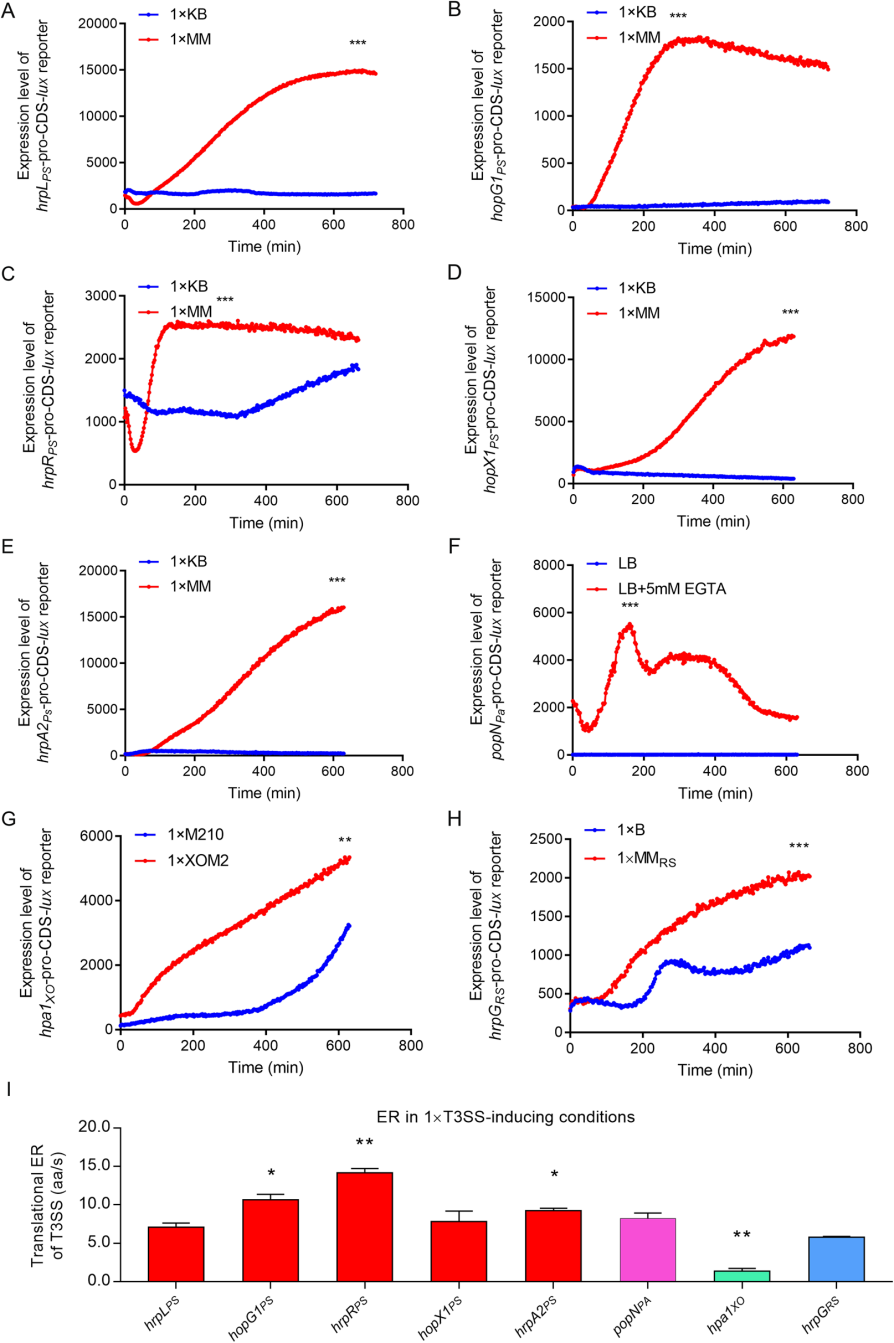
The expression levels of mentioned T3SS proteins were significantly induced in nutrition-limited medium. **A–H** The expression level of T3SS regulators, effectors and structural protein in four model pathogenic bacteria were measured using the transcriptional and translational fusion reporters in 1× T3SS-inducing media and 1× T3SS-suppressing media, including HrpL_PS_*,* HopG1_PS_*,* HrpR_PS_*,* HopX1_PS_*,* HrpA2_PS_*,* PopN_PA_*,* Hpa1_XO_*,* and HrpG_RS_. **I** The translational ERs of the tested T3SS regulators, effectors and structural protein in 1× T3SS-inducing media and 1× T3SS-suppressing media. *p < 0.05; **p < 0.01; and ***p < 0.001. Results were indicated in mean ± SD. All experiments were repeated at least three times

To test the expression pattern of key T3SS regulators, effectors and structural protein in the four above mentioned pathogenic bacteria under T3SS-inducing (1× MM for PS, LB+ 5 mM EGTA for PA, 1× XOM2 for XO and 1× MM_RS_ for RS) and T3SS-suppressing conditions (1× KB for PS, LB for PA, 1× M210 for XO and 1× B for RS), we constructed eight transcriptional and translational fusion reporters containing the full-length CDS of the following T3SS genes: *hrpL*_*PS*_*, hopG1*_*PS*_*, hrpR*_*PS*_*, hopX1*_*PS*_*, hrpA2*_*PS*_*, popN*_*PA*_*, hpa1*_*XO*_ and *hrpG*_*RS*_. As shown in Fig. 1, among these four pathogenic bacteria, T3SS genes were significantly induced by 4–80-fold in the respective T3SS-inducing media but were suppressed in the nutrient-rich media. The expression levels of HrpR_PS_ and PopN_PA_ increased rapidly within the first 3 h in T3SS-inducing conditions (Fig. 1C and F). In contrast, the expression levels of HrpL_PS_*,* HopG1_PS_*,* HopX1_PS_, HrpA2_PS_, Hpa1_XO_ and HrpG_RS_ increased slowly and gradually reached the highest levels within 6–10 h (Fig. 1A, B, D, E, G and H). In addition, we found that the expression levels of HrpL_PS_*,* HrpR_PS_*,* HopX1_PS_ and PopN_PA_ significantly declined in the lag phase, which might be attributed to the bacteria adapting to the altered nutritional environment (Fig. 1A, C, D and F). As negative controls, non-T3SS proteins (alcohol dehydrogenase, AdhB_PS_, lysis phenotype activator, AlpA_PA_, exopolysaccharide xanthan biosynthesis export protein, GumB_XO_, succinyl-CoA synthetase, SucC_RS_ and) were induced to a high level in the nutrient-rich media (Additional file 1: Fig. S1A–D). To investigate if these perceptions can apply to the clinical isolates, we also detected the expression levels of *popN* in *P. aeruginosa* UCBPP-PA14 (*popN*_*PA14*_), a highly virulent and infectious strain [76]. Like PopN_PA_, PopN_PA14_ was significantly induced by threefold in LB with 5 mM EGTA within the first 3 h, and gradually reduced within the following 7 h (Additional file 1: Fig. S1E). Non-T3SS peotein RND multidrug efflux membrane fusion protein, MexA_PA14_, showed similar trend with that of AlpA_PA_ (Additional file 1: Fig. S1F). For HrpG_RS_, the expression in MM_RS_ at 50 and 60 min were induced rapidly so that the synthesis time of HrpG_RS_ was not calculated (Additional file 1: Fig. S1G). Taken together, these results indicate that the expression levels of these T3SS regulators, effectors and structural protein showed similar induction patterns with slight differences in the response time in the T3SS-inducing media. The translational ERs of HrpL_PS_*,* HopG1_PS_*,* HrpR_PS_, HopX1_PS_ and HrpA2_PS_ under 1× T3SS-inducing conditions ranged from 8 to 16 aa/s (Fig. 1I). The ERs of PopN_PA_*,* Hpa1_XO_ and HrpG_RS_ were ~ 9 aa/s, ~ 1.7 aa/s and ~ 6 aa/s, respectively (Fig. 1I). In general, the expression levels of T3SS regulators, effectors and structural protein in these four pathogenic bacteria showed different translational ERs in the T3SS-inducing media.

### Nutrient level and culture duration are negatively correlated with the translational ER of T3SS regulators, effectors and structural protein

**Fig. 2:**
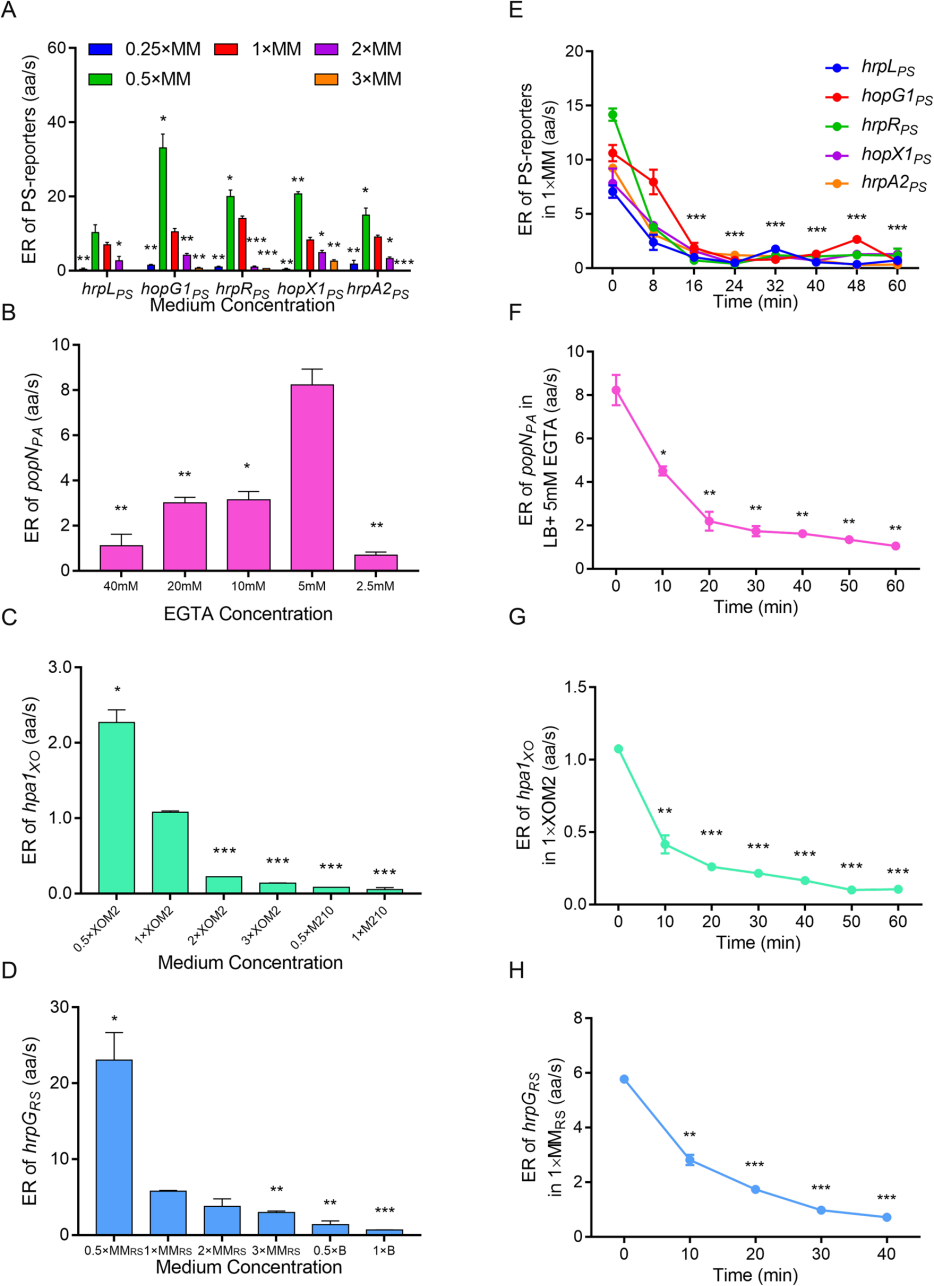
Translational ERs of mentioned T3SS proteins were dependent on the nutrient doses and culture time. **A–H** Translational ERs of T3SS regulators, effectors and structural protein (HrpL_PS_*,* HopG1_PS_*,* HrpR_PS_*,* HopX1_PS_*,* HrpA2_PS_*,* PopN_PA_*,* Hpa1_XO_*,* and HrpG_RS_) under different nutrition-limited conditions and at various culture time (0–60 min). The standard deviations were shown but were very small in the plot. *p < 0.05; **p < 0.01; and ***p < 0.001. Results were indicated in mean ± SD. All experiments were repeated at least three times

Because the translational ERs of the T3SS regulators, effectors and structural protein differed among the tested proteins and strains, we hypothesized that the translational ERs might be influenced by the nutritional conditions and culture durations. To quantify the effect of the nutritional condition on the translational ERs, we first evaluated the translational ERs under nutritional conditions that were lower (0.25× and 0.5× T3SS-inducing medium) and higher (2× and 3× T3SS-inducing medium, and 0.5× rich medium) than the normal T3SS-inducing medium (1× T3SS-inducing medium). The results showed that the T3SS translational ERs of PS, XO and RS in 0.5× MM or 0.5× XOM2 were higher (1.5–3-fold for PS, 1.7-fold for XO and fourfold for RS) than those in 1× MM or 1× XOM2 (Fig. 2A, C and D). Although T3SS protein synthesis in 0.5× T3SS-inducing medium was slightly faster than that in 1× T3SS-inducing medium, the expression levels of T3SS regulators, effectors and structural protein in 0.5× T3SS-inducing medium rapidly decreased (Additional file 1: Fig. S2). For PA, the T3SS translational ERs were calculated in LB broth supplemented with different concentrations of EGTA (40 mM, 20 mM, 10 mM, 5 mM and 2.5 mM) to generate a calcium gradient. The maximum translational ER of PopN_PA_ protein occurred at 5 mM EGTA (Fig. 2B). In general, the T3SS translational ERs of these four bacteria dramatically decreased with increasing nutrient levels in the media.

To investigate the influence of culture duration on the T3SS translational ERs, we measured the translational ERs over the first 60 min in 1× T3SS-inducing media. In PS, the translational ERs of T3SS regulators, effectors and structural protein gradually decreased (~ 90%) from 0 to 16 min after induction and then kept at a low level, showing a time-course behaviour (Fig. 2E). The translational ERs of T3SS regulators and effectors in PA, XO and RS were similar to those in PS (Fig. 2E–H). For PA14, the translational ER of PopN_PA14_ distinctly decreased with the EGTA concentration and culture duration (Additional file 1: Fig. S3A, B). Regarding the translational ERs of non-T3SS proteins (negative controls), the MexA_PA14_ ERs had no significant difference under different EGTA concentrations and at various culrure duration (Additional file 1: Fig. S3C–E). The translational ER of AdhB_PS_ under nutrient-rich conditions (~ 8.5 aa/s) was higher than that in the 2× rich medium and nutrient-limiting medium (less than 1.5 aa/s) (Additional file 1: Fig. S3F). Moreover, the translational ER of AdhB_PS_ in KB at 0 min was eightfold higher than at other points (less than 1.0 aa/s), and all the AdhB_PS_ ERs in MM were low, with no significant differences among them (0.16–0.39 aa/s) (Additional file 1: Fig. S3G, H). These results indicate that the AdhB_PS_ ER was not correlated with culture duration. For PA, the ERs of AlpA_PA_ in LB (~ 13.5 aa/s) were also higher than those found in medium supplemented with EGTA (0.4–3.5 aa/s) (Additional file 1: Fig. S3I). The AlpA_PA_ ERs in LB at 0–40 min (greater than 10 aa/s) were higher than those at 50 and 60 min (~ 2.1 aa/s and ~ 4.0 aa/s, respectively), and were less than 1.0 aa/s in LB supplemented with EGTA (Additional file 1: Fig S3J and K). For XO, the translational ER of GumB_XO_ in 1× M210 (~ 0.5 aa/s) had no significant differences with those in 0.5× M210 and 0.125× M210 (~ 0.8 aa/s). The ERs at 10, 30, 40 and 50 min (~ 0.7–0.8 aa/s) showed no differences with that at 0 min (~ 0.5 aa/s) apart from those at 20 and 60 min (~ 0.9–1.0 aa/s) (Additional file 1: Fig. S3L and M). Similarly, for RS, the translational ERs of SucC_RS_ in 0.5×, 0.25× and 0.125× B (~ 0.9–1.4 aa/s) were much lower than that in 1× B (~ 13.4 aa/s). The translational ERs for 10–60 min (~ 1.3–2.7 aa/s) were much lower than that at 0 min (~ 13.4 aa/s) (Additional file 1: Fig. S3N and O). In general, the translational ERs of T3SS regulators, effectors and structural protein were negatively correlated with the nutrient level and culture duration.

### Translational ERs of T3SS regulators, effectors and structural protein were independent on the mRNA transcription

**Fig. 3:**
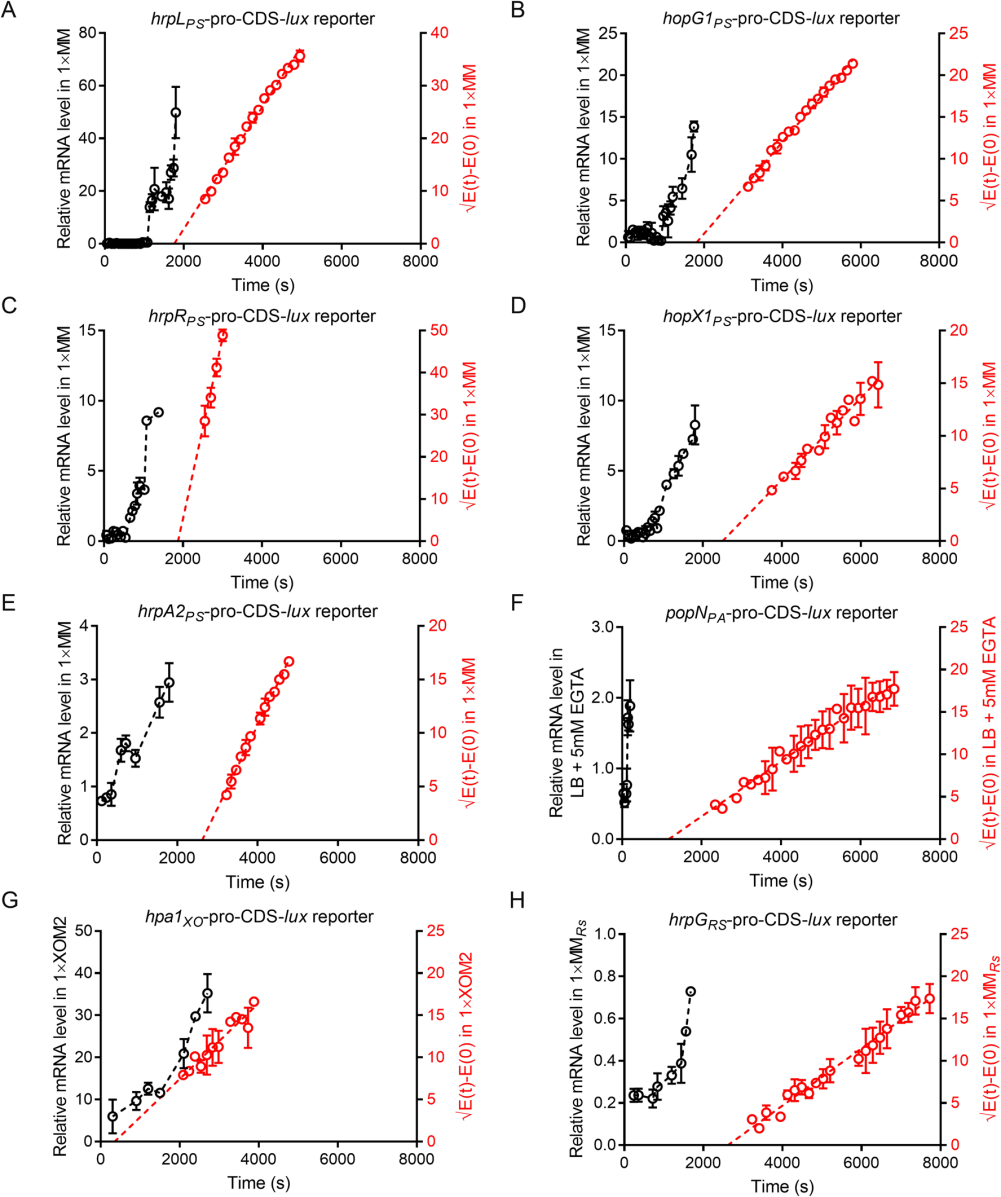
The first T3SS full-length protein synthesis back the full-length mRNA synthesis under 1× T3SS-inducing conditions. **A–H** Induction assay of full-length T3SS mRNAs (*hrpL*_*PS*_*, hopG1*_*PS*_*, hrpR*_*PS*_*, hopX1*_*PS*_*, hrpA2*_*PS*_*, popN*_*PA*_*, hpa1*_*XO*_ and *hrpG*_*RS*_) of transcriptional and translational fusion reporters which were induced in 1× T3SS-inducing conditions. The qRT-PCR primers were used to detect the 3′ end region of T3SS mRNAs. qRT-PCR detected the synthesis kinetics of full-length T3SS mRNAs. The Schleif plots ($\sqrt{E\left(t\right)-E(0)}$) of all T3SS mRNAs were shown in red. The standard deviations were shown but were very small in the plot. All experiments were repeated at least three times

The first step of translational elongation is the recognition and regulation of cognate tRNAs, which is carried out by mRNA codons [8]. The mRNA synthesis levels can change in accordance with the cell cycle [77, 78]. To investigate the influence of mRNA synthesis on the translational ER of T3SS regulators, effectors and structural protein, we assessed the induction kinetics of T3SS gene mRNAs in the transcriptional and translational fusion reporters of four pathogenic bacteria over the first 30 min in T3SS-inducing medium using qRT-PCR with reverse primers at the 3′ end. As shown in Fig. 3, the expression levels of full-length T3SS mRNAs were strongly induced under nutrient-limiting conditions. The initial synthesis time of these T3SS regulators, effectors and structural protein showed similar patterns of requiring approximately 2000 s (red lines in Fig. 3). However, the transcription durations of the T3SS mRNAs showed different kinetics trends. Among them, *hrpL*_*PS*_ and *hopG1*_*PS*_ mRNA transcription required ~ 1080 s and ~ 900 s, which were shorter than the initial first full-length protein syntheses (~ 1769 s and ~ 1830 s, respectively) (Fig. 3A and B). The mRNA synthesis durations of other T3SS regulators, effectors and structural protein were less than ~ 540 s (Fig. 3C–H). Notably, the full-length mRNAs of *hrpA2*_*PS*_, *popN*_*PA*_ and *hpa1*_*XO*_ were rapidly synthesized (~ 480 s, ~ 140 s and ~ 300 s) (Fig. 3E–G), while the first full-length protein syntheses began at ~ 2485 s, ~ 1178 s and ~ 350 s, respectively. For clinical strain, the transcription level of *popN*_*PA14*_ mRNA was rapidly induced at ~ 360 s in LB with 5 mM EGTA, much shorter than the initial first full-length PopN_PA14_ syntheses (~ 2191 s) (Additional file 1: Fig. S4A). For non-T3SS proteins, the *adhB*_*PS*_ mRNA level in MM gradually declined throughout 7 h of culture, while the translation duration of AdhB_PS_ was longer than 5 h (Additional file 1: Fig. S4B). In contrast, in PA and PA14, the full-length mRNA of *alpA*_*PA*_ and *mexA*_*PA14*_ were rapidly synthesised (~ 15 min and ~ 10 min), leading to their translation at ~ 47.8 min and ~ 4.9 min (Additional file 1: Fig. S4C and D). These results indicate that protein synthesis began when the full-length mRNA level of T3SS genes was sufficiently high, demonstrating that under nutrient-limiting conditions, the translational ERs of T3SS regulators, effectors and structural protein were independent on the mRNA synthesis levels.

### Cellular tRNA levels positively affect the translational elongation of T3SS proteins in the latter culture period

**Fig. 4:**
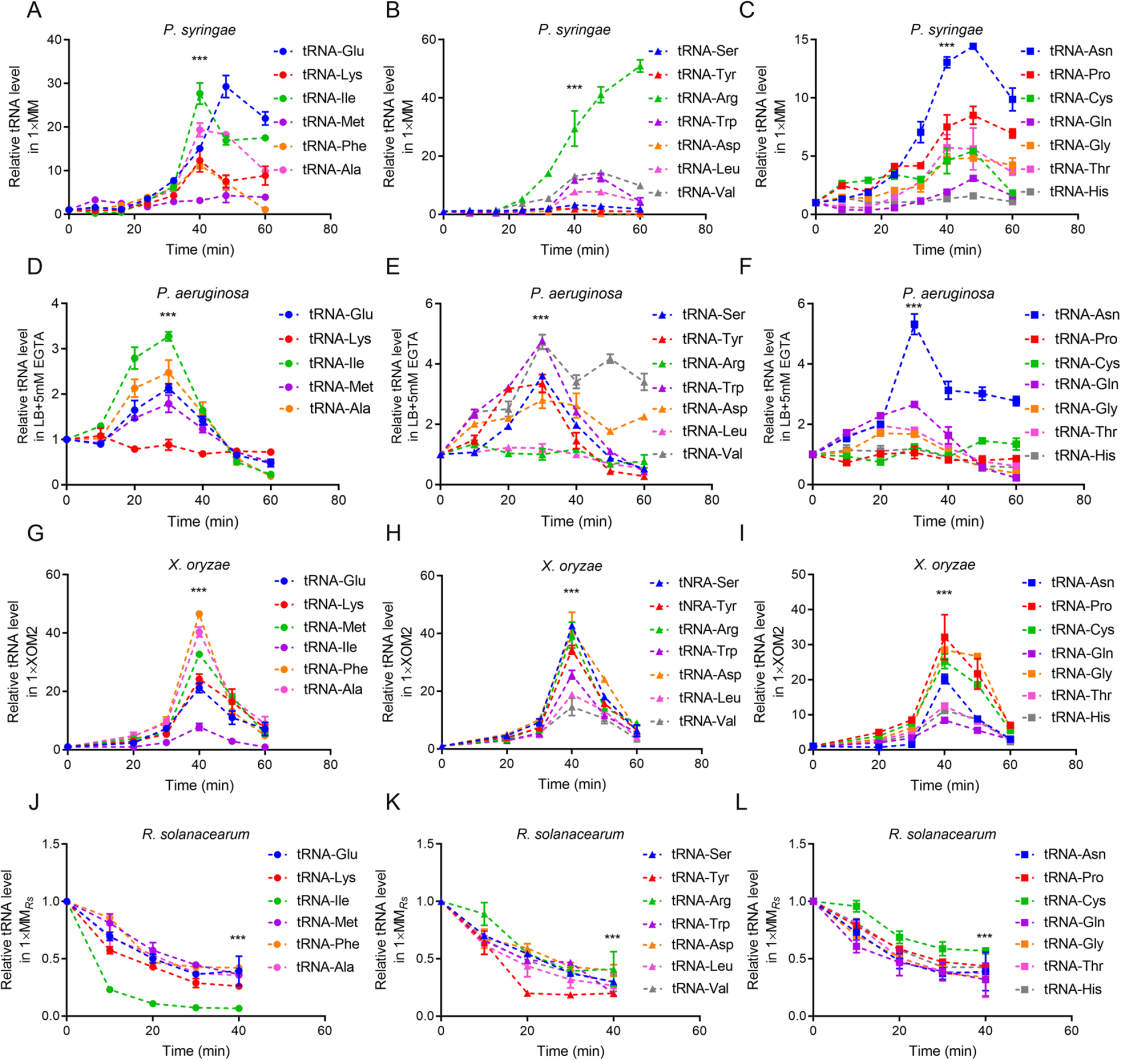
Translational ERs of mentioned T3SS proteins were limited by total tRNA pools at later phase. **A–C** Twenty different tRNAs of four mentioned bacteria were detected by qRT-PCR with random primers. The cellular total tRNAs levels at various culture times in 1× T3SS-inducing conditions. The tRNAs of PS were detected at 8, 16, 24, 32, 40, 48, 60 min. **D–L** tRNAs of PA, XO and RS were detected at 10, 20, 30, 40, 50, 60 min. All experiments were repeated at least three times. The data at 0 min for each individual tRNA was set as 1. *p < 0.05; **p < 0.01; and ***p < 0.001. Results were indicated in mean ± SD. **A**–**C** showed tRNA-Ile, tRNA-Arg and tRNA-Asn as represents of *P. syringae*. **D**–**F** showed tRNA-Ile, tRNA-Val and tRNA-Asn as represents of *P. aeruginosa*. **H**–**J** showed tRNA-Phe, tRNA-Asp and tRNA-Gly as represents of *X. oryzae*. **K**–**M** showed tRNA-Ile, tRNA-Tyr and tRNA-His as represents of *R. solanacearum*. The standard deviations were shown but were very small in the plot

Because the translational ER is also related to cellular tRNA levels [1], we hypothesized that the reduced translational ERs during the first 60 min of culture might be caused by the tRNA levels. To verify this hypothesis, we quantitatively characterised the tRNA levels of transcriptional and translational fusion reporters of the four tested bacteria using the different culturing conditions and durations. Most of the 20 tested tRNAs displayed a similar tendency. As expected, the tRNA levels of RS were decreased by ~ 40% at 40 min compared with those at 0 min (Fig. 4J–L), which correlated with the reduced translational ERs of HrpG_RS_ (Fig. 2H). The tRNA-Ile level had rapidly decreased by ~ 80% at 10 min and by ~ 90% at 20 min (Fig. 4J). Compared with 0 min, the tRNA-Tyr level at 20 min had declined by ~ 80% (Fig. 4K).

The tRNA expression patterns showed different trends in other bacteria. In PS, although the tRNA expression levels significantly increased with the culture duration under T3SS-inducing conditions, the expression of most tested tRNAs had decreased after 48 min (Fig. 4A–C). The tRNA-Glu and tRNA-Asn expression levels were greater than tenfold higher than those at the beginning of induction, and the level of tRNA-Arg was 50-fold higher than that at 0 min. We also found that the expression levels of tRNA-Ile, tRNA-Ala and tRNA-Lys had declined at 40 min. The expression levels of tRNA-Met, tRNA-Tyr, tRNA-Ser and tRNA-His showed no significant variations throughout the induction. For PA, the expression levels of most tRNAs peaked at 30 min and then decreased (Fig. 4D–F). The levels of tRNA-Trp, tRNA-Val and tRNA-Asn increased by ~ fivefold (Fig. 4E and F). Unlike other tRNAs that decreased to their initial level at 60 min, the expression levels of tRNA-Val, tRNA-Asp and tRNA-Asn at 60 min were ~ two–threefold higher than those at 0 min (Fig. 4E and F). However, the expression levels of tRNA-Lys, tRNA-Arg, tRNA-Leu, tRNA-Pro, tRNA-Cys and tRNA-His were stable throughout the 60-min culture. For PA14, most of the 20 tested tRNAs represented the highly increase within the first 20–30 min and the gradually decline in the following 30–40 min, like tRNA-Ile (Additional file 1: Fig. S5A-C). For XO, tRNA expression peaked at 40 min (Fig. 4G–I), followed by a rapid decrease to the initial levels by 60 min. Taken together, these results indicated that the tRNA expression levels showed trends similar to those of the corresponding translational ERs under T3SS-inducing conditions in the latter culture period.

### EFs positively regulate the translational elongations of T3SS regulators, effectors and structural protein

**Fig. 5:**
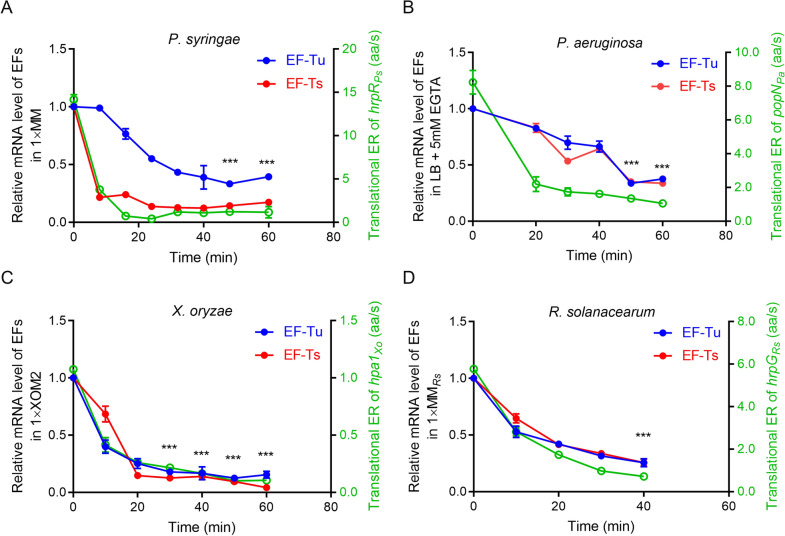
The expression of EF-Tu and Ts decreased with the culture time. **A–D** The relative EFs levels of four above mentioned bacteria were detected by qRT-PCR under the same nutrition conditions and times as Fig. 2E–H. The EFs levels were shown together with the translational ER of related strain in 1× T3SS-inducing conditions. The translational ERs at specific times were shown as green hollow symbols. The data at 0 min for each individual EF was set as 1. The standard deviations were shown but were very small in the plot. *p < 0.05; **p < 0.01; and ***p < 0.001. Results were indicated in mean ± SD. All experiments were repeated at least three times

EF-Tu and EF-Ts play critical roles in polypeptide elongation [79]. To further understand the translational elongation patterns of T3SS regulators, effectors and structural protein in the tested pathogenic bacteria, we detected the expression levels of EF-Tu and EF-Ts under the different culture conditions and at various culture durations. In PS (Fig. 5A), the EF-Tu level gradually declined and at 60 min was reduced by ~ 40% compared with 0 min. Compared with EF-Tu, at 8 min, the expression level of EF-Ts was decreased by ~ 85%. Similar to the HrpR_PS_ translational ER (solid green line in Fig. 5A), EF-Ts expression level remained low throughout the 16–60 min of culture. In XO, EF-Tu expression level was reduced by ~ 60% at 10 min and by ~ 85% at 60 min, while the expression level of EF-Ts was decreased by ~ 40% at 10 min and by ~ 90% at 60 min.

In contrast, in XO, the reduced translational ER of Hpa1_XO_ was more consistent with the EF-Tu level (Fig. 5C). In RS, the trends in these two EF levels were similar, showing an ~ 75% decrease at 60 min, which was similar to the trend for the HrpG_RS_ translational ER. We also found that the translational ERs of PS, PA and RS decreased to a greater extent (by ~ 90%) than the respective EFs (by ~ 60%) in nutrient-limiting conditions (Fig. 5A, B and D). Different from relative expression levels of EFs of the model pathogenic bacteria at 10 min (less than 1), the relative expression levels of EFs of PA14 were high up to 7. Even at 40 min, the relative expression level of EF-Tu was still as high as 1.6. The high expression of EFs of PA14 provided support to the fast translational ER and conduced to the high virulence (Additional file 1: Fig. S5D). The translational ERs of these five bacteria decreased by more than 90% after 60 min of culture, while the EF levels had reduced by 70–90% at 60 min (Fig. 5 and Additional file 1: Fig S5). Overall, similar trends in the translational ERs and EF levels indicated that EF-Tu and EF-Ts play important roles on the translational elongation of T3SS regulators, effectors and structural protein under nutrient-limiting conditions.

## Discussion

Rapid T3SS protein synthesis is vital for the invasion of pathogenic bacteria. However, the patterns of T3SS protein translation have not been elucidated. In prokaryotes, variations in *E. coli* translational elongation have been studied [80–83]. Initiation of mRNA translation, but not the concentration of tRNAs, is a rate-limiting step for protein synthesis under nutrient-rich conditions [16, 84]. In environments with low carbon and nitrogen availability, the ERs in *E. coli* gradually decrease, but they remain constant in rich conditions [74, 81, 85]. In contrast, our data suggested that the translational ERs of T3SS in pathogenic bacteria steadily declined with increasing nutrient concentrations and culture time. Although maintaining a low ER in the later period of incubation, T3SS proteins gradually accumulated. Rapid ER of T3SS proteins in the early incubation reflected the instantaneous response of pathogenic bacteria to environmental change. In addition, we demonstrated that the T3SS translational ERs decreased with the prolongation of culture duration. These results indicate that both the total tRNA and the EF levels are constraints on the translational elongation of T3SS regulators, effectors and structural protein. In the latter culture period, the degradation of total tRNAs and the gradual reduction of EF levels restricted the T3SS translational ERs. These findings reveal key aspects of T3SS translational ERs in pathogenic bacteria under nutrient-limiting conditions.

The results showed that the reduced translational ERs were caused by decreasing tRNA levels in the latter culture period (Fig. 4). From 30–40 to 60 min of culture, the reduction in tRNAs was consistent with the decrease in translational ERs (Fig. 2E–H). A model of tRNA degradation suggests that tRNAs degrade with early amino acid starvation in *E. coli*, which is in-line with the reduced demand for protein and the improved quality in protein synthesis [86]. In addition, the charge level of tRNAs rapidly decreases during early starvation and the reduced number of charged tRNAs inhibits the translational elongation of T3SS. In the present study, total tRNAs in PS, PA and XO quickly accumulated from 0–30 to ~ 40 min of culture. Studies have shown that tRNA accumulation results in cells death [87]. Accumulating tRNAs can result in the incorrect insertion of an amino acid into a polypeptide, leading to a decrease translational ER [88]. As mRNA transcription also plays an important role on the T3SS expression under nutrient starvation, we calculated the transcriptional ERs of T3SS regulators, effectors and structural protein in the four mentioned pathogenic bacteria (Table 1). Compared with the translational ERs, the transcriptional ERs were much lower, indicating the significant effect of translational ER on T3SS protein synthesis.

**Table 1:** T3SS transcriptional ERs were much lower than the T3SS translational ERs in 1× T3SS-inducing media

Tested proteins	Transcriptional ER (3 nt/s)	Translational ER (aa/s)
HrpL_PS_	0.17 ± 0.01	7.08 ± 0.57
HopG1_PS_	0.56 ± 0.03	10.63 ± 0.74
HrpR_PS_	0.54 ± 0.04	14.18 ± 0.56
HopX1_PS_	0.67 ± 0.05	8.41 ± 0.55
HrpA2_PS_	0.28 ± 0.03	9.24 ± 0.33
PopN_PA_	2.01 ± 0.57	8.24 ± 0.7
Hpa1_XO_	0.43 ± 0.05	1.08 ± 0.02
HrpG_RS_	0.64 ± 0.04	5.78 ± 0.12

In Table 1, we defined the time of mRNA production as *T*_*mRNA*_ and unified the unit as 3 nt/s. Therefore, the transcriptional ER was calculated as *L*_*m*_*/3T*_*mRNA*_, which *L*_*m*_ was the length of each full-length mRNA. Results are indicated in mean ± SD. All experiments were repeated at least three times.

In addition to increasing tRNA levels, we found that reduced EF levels also resulted in decreased translational ERs of T3SS regulators, effectors and structural protein under nutrient-limiting conditions. Charged tRNAs move to the ribosome as a ternary complex with GTP and EF-Tu; this complex can be hydrolyzed and released as EF-Tu–GDP [89]. GDP is reactivated to GTP through a sequence of nucleotide exchanges carried out by EF-Tu–Ts complexes [79]. Our results showed that the EF levels had rapidly decreased by ~ 60% after 40 min in nutrient-limiting conditions (Fig. 5), suggesting their positive regulation of the T3SS translational ERs. The ribosome concentration is strongly affected by the EF-Ts concentration whereby a high EF-Ts level facilitates rapid cell growth [90, 91]. Therefore, the reduced EF levels of the four bacteria suggest that few ternary complexes participate in T3SS translation under nutrient-limiting conditions, causing the decreased translational ERs (Fig. 5).

To better understand the virulence of the tested pathogens, we detected the relative expression level of the tested T3SS genes during the first 0–6 h after induction. As shown in Additional file 1: Fig. S6A-E, the expression levels of *hrpL*_*PS*_, *hpa1*_*XO*_ and *hrpG*_*RS*_ markedly increased during 0–6 h-induction. For *popN*_*PA*_ and *popN*_*PA14*_, the expression levels enhanced during 0-3 h and then gradually reduced in the following 3 h. As a repressor of T3SS, the decrease of the transcription of *popN* contributed to the invasion of T3SS to the host. The qRT-PCR result revealed the dynamic change of T3SS genes after induction.

**Fig. 6:**
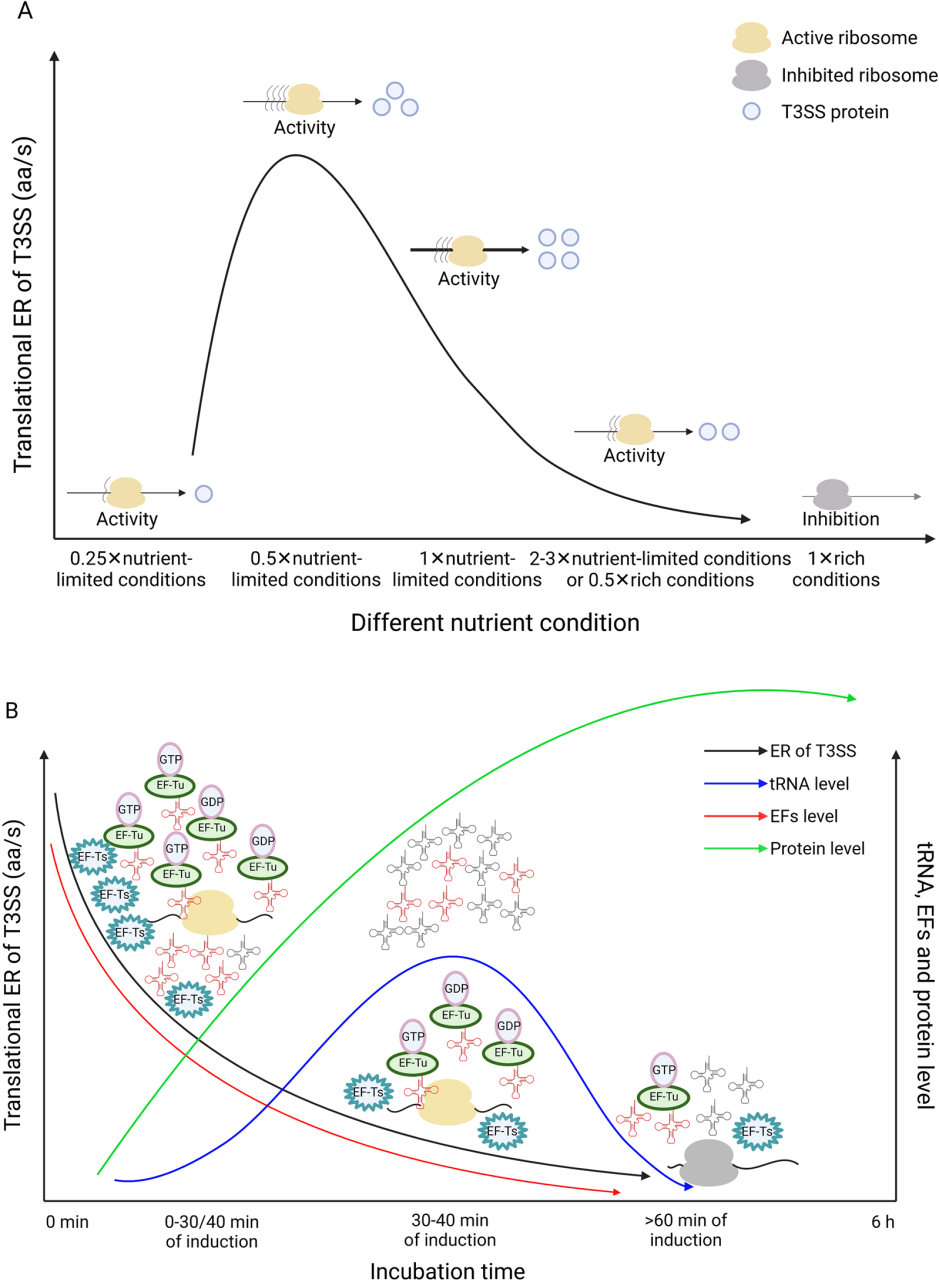
Schematic diagram of translational ER and protein synthesis of T3SS under nutrition-limited conditions. **A** Translational ER was negatively correlated with nutrition conditions. Under rich nutrition conditions, translational ER of T3SS protein was almost completely ceased. Under 0.5× rich conditions, 3× and 2× T3SS-inducing conditions, translational ER of T3SS protein increased but at a low level. Under 1× T3SS-inducing conditions, translational ER was high to support the efficient protein synthesis. Under 0.5× T3SS-inducing conditions, translational ER was higher than that under 1× T3SS-inducing conditions, but the final protein production was unstable. Under 0.25× T3SS-inducing conditions, translational ER dropped by 95%. **B** Translational ER was negatively correlated with culture time under 1× T3SS-inducing conditions. At 0–30/40 min of induction, reduced EF-Tu, Ts and incorrect insertions of amino acid caused by increased charged tRNAs led to the descending translational ER of T3SS. At 30/40–60 min of induction, degradation of tRNAs and reduced EF-Tu and Ts resulted in the descending translational ER of T3SS. The expression levels of T3SS regulators, effectors and structural protein gradually increased within 0–6 h of induction

Based on the results of this study, we propose a model in which the levels of tRNAs and EFs regulate translational ERs under nutrient-limiting conditions. The translational ERs were negatively correlated with the nutrient concentration. As shown in Fig. 6A, the expression level of T3SS regulators, effectors and structural protein was low under nutrient-rich conditions. Under 0.5× nutrient-rich conditions and 3× and 2× T3SS-inducing conditions, the T3SS translational ERs slightly increased but remained low. In the 1× T3SS-inducing condition, the T3SS translational ERs rapidly increased and supported a high amount of protein synthesis. Under the 0.5× T3SS-inducing condition, the translational ERs were higher than those under the 1× T3SS-inducing condition, but the final amount of protein production varied. Under the 0.25× T3SS-inducing condition, the translational ERs rapidly decreased. Figure 6B shows that the incubation duration negatively regulated the translational ERs under the 1× T3SS-inducing condition. From 0–30 to ~ 40 min of induction, the decreasing T3SS translational ERs were caused by the reduced EF levels. From 30–40 to 60 min of induction, tRNAs degradation and reduced EF levels resulted in decreasing T3SS translational ERs. The expression level of T3SS regulators, effectors and structural protein gradually increased within 6 h of induction and was then stably maintained.

In conclusion, our study elucidated that tRNAs and EFs play important roles in regulating the translational ERs of T3SS regulators, effectors and structural protein. We characterized the T3SS translational ERs based on a luminescence reporter system, which quantitatively illustrated the translational elongation pattern of T3SS under nutrient-limiting conditions. Different but fast ERs of various T3SS proteins in the early incubation numerically emphasize the multiple response of pathogenic bacteria to environmental change. Our findings provide insights into the quantitative translational of T3SS and the significance of tRNAs, EF-Tu and EF-Ts on T3SS translational elongation under nutrient-limiting conditions. These findings in four model pathogenic bacteria might apply to other bacterial species.

## Experimental procedures

### Bacterial strains, plasmid and primers

The bacterial strains used in this study were wild-type *P. syringae* (PS) 1448A, *P. aeruginosa* (PA) PAO1, *P. aeruginosa* UCBPP-PA14 (PA14) PA14, *X. oryzae* (XO) PXO99A and *R. solanacearum* (RS) OE 1-1. The tested genes were *hrpL*_*PS*_, *hopG1*_*PS*_, *hrpR*_*PS*_, *hopX1*_*PS*_, *hrpA2*_*PS*_, *adhB*_*PS*_, *popN*_*PA*_, *alpA*_*PA*_, *popN*_*PA14*_, *mexA*_*PA14*_, *hpa1*_*XO*_*, gumB*_*XO*_, *hrpG*_*RS*_* and sucC*_*RS*_. The pMS402 plasmid was used [34], which encodes a promoter-less luciferase (*lux*). The bacterial strains, plasmid and primers are listed in Table S1 in the Additional file 1. Each T3SS gene included two reporters. One included the corresponding promoter and a promoter-less luciferase (*lux*) in the plasmid, which was the transcriptional fusion reporter. The other reporter concluded the corresponding promoter, the coding sequence (CDS) and a promoter-less luciferase (*lux*) in the plasmid, which was the transcriptional and translational fusion reporter.

### Cell growth conditions

PS 1448A, XO PXO99A and RS OE 1-1 were grown in the nutrient-rich media King’B (KB) [92], M210 [93] and B [94], respectively at 28℃ until reaching an optical density at 600 nm (OD_600_) of ~ 0.6. PA PAO1 and PA14 were cultured in LB at 37 °C. For PS, XO and RS, the pre-culture was centrifuged, washed three times, and resuspended in distilled water. For the induction curve, the *lux* activity of the final experimental culture at OD_600_ ~ 0.1 in the T3SS-inducing condition was immediately measured. The nutrient-limiting media were MM for PS (50 mM KH_2_PO_4_, 7.6 mM (NH_4_)_2_SO_4_, 1.7 mM MgCl_2_, 1.7 mM NaCl, and 10 mM fructose, pH 5.7) [95], XOM2 for XO (0.18% xylose sugar, 670 μM D, L-methionine, 10 mM sodium L(+)-glutamate, 14.7 mM KH_2_PO_4_, 40 μM MnSO_4_, 240 μM Fe(III) EDTA and 5 mM MgCl_2_, pH 6.5) [96] and MM_RS_ for RS [97], respectively. To investigate different nutrient conditions, nutrient gradients of the media were used to induce nutrient-limiting conditions. For the PA and PA14 pre-culture, 5 mM EGTA and 100 mM MgCl_2_ were added to induce T3SS gene expression, and various doses of EGTA and MgCl_2_ were used to achieve a range of calcium stress conditions. Related parameters including the translational ER and the mRNA, tRNA and EF levels were measured at specific time points. The kanamycin concentrations were 100 μg/mL for PS and 50 μg/mL for XO and RS. The trimethoprim concentration was 50 μg/mL for PA and PA14.

### Translational ER measurement

The measurement and calculation of the translational ER were based on a luminescence reporter system and a LacZ induction assay, respectively [4, 75]. Therefore, we constructed two kinds of reporters. One included the corresponding gene promoter and a promoter-less luciferase (*lux*) in the plasmid, which was the transcriptional reporter to calculate the transcription time (*T*_*initiation*_). The other reporter concluded the corresponding promoter, the CDS and a promoter-less luciferase (*lux*) in the plasmid, which was the reporter for both transcription and translation to calculate the time for both processes (*T*_*test*_). The synthesis cost of the promoters was assessed as the initiation time, and the associated calibrations are described in Additional file 1: Fig. S7. Immediately after transferring into the investigated media (for PS, XO and RS) or after the addition of EGTA and MgCl_2_ (for PA and PA14), the *lux* activity was measured using a microplate reader (600 nm excitation filter) at 2.5-min intervals. The induction curve of the tested proteins was constructed by plotting the *lux* activity against the induction time and analysed with a Schleif plot. We considered *E(0)* as the basal *lux* activity of the reporter and *E(t)* as the *lux* activity at the specific time point of induction. $\sqrt{E\left(t\right)-E(0)}$ is positively correlated with time [98]; therefore, the x-intercept of the Schleif plot is the time needed for translating one molecule. *T*_*test*_ was regarded as the intact cost time, and *T*_*initiation*_ was regarded as the initiation cost time. The synthesis time of the first round of the tested proteins, *T*_*first*_, could thus be calculated as:

1 $$ T_{first} = T_{test} - T_{initiation} $$

Therefore, the translational ER can be calculated as:

2 $$ {\text{ER }} = \, {{\text{L}} \mathord{\left/ {\vphantom {{\text{L}} {\left( {T_{test} - T_{initiation} } \right)}}} \right. \kern-\nulldelimiterspace} {\left( {T_{test} - T_{initiation} } \right)}} $$

where L is the length of each full-length protein.

The $\sqrt{E\left(t\right)-E(0)}$ values of Hpa1_XO_ and HrpG_RS_ were showed in Additional file 1: Fig. S8.

### Measuring mRNA, tRNA and EF abundances by qRT-PCR

The mRNA, tRNA and EF abundances were measured by quantitative reverse transcription (qRT-PCR). The qRT-PCR primers used are shown Table S1 in the Additional file 1. To measure the transcriptional kinetics of the full-length T3SS mRNAs, the transcriptional and translational fusion reporters were grown to OD_600_ ~ 0.6 and then transferred into the investigated media. Immediately after induction, 500 μL of culture was removed at 1-min intervals and added to 500 μL of pre-cooled stop solution containing 60% ethanol, 2% phenol and 10 mM EDTA. The total RNA purification of PS, PA, PA14 and XO was performed with a RNeasy mini kit (Qiagen); the RS total RNA was isolated using TRIzol reagent (Invitrogen). The RNA concentrations were measured using a NanoDrop 2000 spectrophotometer (ThermoFisher). cDNA synthesis was performed using HiScript III RT SuperMix (Vazyme, China). qRT-PCR was performed with a SuperReal Premix Plus (SYBR Green) kit (Tiangen Biotech) according to the manufacturer’s instructions. The relative mRNA abundance at each time point was 2^Ct(0)−Ct(t)^, where *Ct (0)* indicates the Ct value of the sample taken immediately before transfer to nutrient-limiting conditions, and *Ct (t)* indicates the Ct value at each time point thereafter. The relative mRNA synthesis level was plotted against time to obtain the transcriptional kinetics curve.

To measurement the relative tRNA and EF levels, 500 μL of culture was removed at 8-min (PS) or 10-min (PA, PA14, XO and RS) intervals and centrifuged at 12,000 rpm for 1 min to harvest the bacteria. The RNA purification and reverse transcription processes were performed as previously mentioned, except the total RNA was incubated at 80 °C for 15 min before cDNA synthesis to remove the secondary structure of tRNA. The corresponding 16S rRNAs of PS, PA and PA14, *gyrB* of XO and *serC* of RS were used as internal references. Relative expression levels of tRNAs and EFs at various time points were calculated by the 2^−(ΔΔCt)^ method.

## Supplementary Information

**Additional file 1: Figure S1.** Induction curves of PopN_PA14_ and non-T3SS proteins (AdhB_PS_, AlpA_PA_, MexA_PA14_, GumB_XO_ and SucC_RS_) induced in 1× T3SS-inducing conditions and 1× T3SS-repressing conditions. (**A**) The red curve showed the delayed expression levels of *adhB*_*PS*_-pro-CDS-*lux* reporter in 1× MM compared with that in 1× KB. (**B**) The red curve showed the slightly fast expression levels of *alpA*_*PA*_-pro-CDS-*lux* reporter in LB + 5 mM EGTA compared with that in LB. (**C**) The red curve showed the delayed expression levels of *gumB*_*XO*_-pro-CDS-lux reporter in 1× XOM2 compared with that in 1× M210. **(D)** The red curve showed the delayed expression levels of *sucC*_*RS*_-pro-CDS-lux reporter in 1× MM_RS_ compared with that in 1× B. **(E)** The red curve showed the slightly fast expression levels of *popN*_*PA14*_-pro-CDS-*lux* reporter in LB + 5 mM EGTA compared with that in LB. (**F**) The red curve showed the slightly fast expression levels of *mexA*_*PA14*_-pro-CDS-*lux* reporter in LB + 5 mM EGTA compared with that in LB. (**G**) The expression of HrpG_RS_ in MM_RS_ at 50 and 60 min were induced rapidly so that the synthesis time of HrpG_RS_ was not calculated. **Figure S2.** Expression levels of T3SS regulators, effectors and structural protein of *P. syringae*, *X. oryzae* and *R. solanacearum* in 0.5× T3SS-inducing conditions were less stable than that in 1× T3SS-inducing conditions. The red curve and green curve denoted the expression levels in 1× and 0.5× T3SS-inducing conditions, respectively. (**A**) The expression levels of *hrpL*_*PS*_-pro-CDS-*lux* reporter were extremely low in 0.5× MM compared with those in 1× MM. (**B-D, F**) The expression levels of *hopG1*_*PS*_, *hrpR*_*PS*_, *hopX1*_*PS*_ and *hrpG*_*RS*_-pro-CDS-*lux* reporters in 0.5× T3SS-inducing conditions rapidly increased, but declined to a low level immediately compared with those in 1× T3SS-inducing conditions. (**E**) The expression levels of *hpa1*_*XO*_-pro-CDS-*lux* reporter in 0.5× XOM2 were slightly lower than those in 1× XOM2. **Figure S3.** Translational elongation rate of PopN_PA14_ and non-T3SS proteins (AdhB_PS_, AlpA_PA_, MexA_PA14_, GumB_XO_ and SucC_RS_) in multiple nutrition conditions and at various time. **(A-B)** Translational ERs of PopN_PA14_ under different EGTA concentrations and at various culture time (0–60 min). (**C**) The translational ERs of MexA_PA14_ in LB with EGTA (~ 1.0- ~ 2.2 aa/s) were much lower than that in LB (~ 5.2 aa/s) with no significant difference. (**D-E**) The translational ERs of MexA_PA14_ in both LB and LB with 5 mM EGTA during various culture times kept at a low level (~ 0.5- ~ 2.0 aa/s and ~ 0.8- ~ 1.8 aa/s, respectively). (**F**) The translational ER of AdhB_PS_ in 1× MM (~ 8 aa/s) was more than fourfold than those in richer or poorer nutrition conditions (less than 2 aa/s). (**G**) In 1× KB, the translational ER of AdhB_PS_ at the beginning of culture (~ 8 aa/s) had significant difference than those at various culture time (less than 2 aa/s). (**H**) In 1× MM, the translational ERs of AdhB_PS_ at 0–4 h of culture were much lower than that in 1× KB, which had no difference. (**I**) The translational ER of AlpA_PA_ in LB (~ 13 aa/s) was more than threefold than those in LB + EGTA (less than 5 aa/s). (**J**) In LB, the translational ERs of AlpA_PA_ at 0–40 min of culture (10–15 aa/s) had no difference. The ER decreased rapidly at 50–60 min (less than 5 aa/s). (**K**) In LB + 5 mM EGTA, the translational ERs of AlpA_PA_ were much lower than those in LB. **(L)** The translational ER of GumB_XO_ in 1× M210 (~ 0.5 aa/s) had no significant differences with those in 0.5× M210 and 0.125× M210 (~ 0.8 aa/s). (**M**) The translational ERs of GumB_XO_ at 10, 30, 40 and 50 min (~ 0.7–0.8 aa/s) showed no differences with that at 0 min (~ 0.5 aa/s) apart from those at 20 and 60 min (~ 0.9–1.0 aa/s). (**N**) The ERs of SucC_RS_ in 0.5× , 0.25× and 0.125× B (~ 0.9–1.4 aa/s) were much lower than that in 1× B (~ 13.4 aa/s). (**O**) The translational ERs of SucC_RS_ for 10–60 min (~ 1.3–2.7 aa/s) were much lower than that at 0 min (~ 13.4 aa/s). **Figure S4.** Induction assay of full-length PopN_PA14_ and non-T3SS proteins (AdhB_PS_, AlpA_PA_ and MexA_PA14_) mRNAs in 1× T3SS-inducing conditions. (**A**) The mRNA synthesis level of *popN*_*PA14*_ was rapidly induced in LB with 5 mM EGTA. $\sqrt{E\left(t\right)-E(0)}$ plot showed that *T*_*test*_ of PopN_PA14_ was ~ 2191 s in LB with 5 mM EGTA. (**B**) The mRNA synthesis level of *adhB*_*PS*_ rapidly decreased in 1× MM and maintained a low level within 26–420 min. $\sqrt{E\left(t\right)-E(0)}$ plot showed that *T*_*test*_ of AdhB_PS_ was ~ 343.7 min in 1× MM. (**C**) The mRNA synthesis level of *alpA*_*PA*_ increased after adding EGTA and dropped to the starting level. $\sqrt{E\left(t\right)-E(0)}$ plot showed that *T*_*test*_ of AlpA_PA_ was ~ 47 s in LB + 5 mM EGTA. (**D**) The mRNA synthesis level of *mexA*_*PA14*_ increased after adding EGTA and dropped to the starting level. $\sqrt{E\left(t\right)-E(0)}$ plot showed that *T*_*test*_ of MexA_PA14_ was ~ 5 min in LB + 5 mM EGTA. **Figure S5.** Relative expression levels of tRNAs and EFs of PA14 in LB with 5 mM EGTA. (**A-C**) tRNAs of PA14 were detected at 10, 20, 30, 40, 50, 60 min. The data at 0 min for each individual tRNA was set as 1. (**D**) The relative EFs levels of PA14 were detected by qRT-PCR. The EFs levels were shown together with the translational ER of PopN_PA14_ in LB with 5 mM EGTA. The translational ERs at specific times were shown as green hollow symbols. The standard deviations were shown but were very small in the plot.  *p < 0.05; **p < 0.01; and ***p < 0.001. Results were indicated in mean ± SD. All experiments were repeated at least three times. **Figure S6.** The expression levels of T3SS genes during 0–6-h inducing culture. (**A–E**) The expression levels of *hrpL*_*PS*_, *popN*_*PA*_, *hpa1*_*XO*_, *hrpG*_*RS*_ and *popN*_*PA14*_ in 1× T3SS-inducing conditions for 0–6 h. **Figure S7.** The Schleif plot of T3SS regulators, effectors and structural protein of *P. syringae*, *P. aeruginosa*, *X. oryzae* and *R. solanacearum* in 1× T3SS-inducing conditions. The Schleif plot was used to detect the translational time of the first newly synthesized protein in 1× T3SS-inducing conditions. The square root of newly synthesized protein ($\sqrt{E\left(t\right)-E(0)}$) was linear correlated with time during the initial dozens of minutes. *E(t)* denoted the expression levels of the tested proteins at specific times in 1× T3SS-inducing conditions, and *E(0)* denoted the basal expression levels of the culture. The green line and red line described the linear line of transcriptional fusion reporters and reporters containing both transcription and translation, respectively. The x-intercepts of the green line and red line denoted the time for the initial cost (*T*_*initiation*_) and the transcription and translation of the tested proteins (*T*_*test*_), respectively. Therefore, the difference of the two intercepts corresponded to the time to translate the full-length protein molecule (*T*_*first*_). The initial cost included the sense of cells to nutrient conditions, RNA polymerase transcriptional initiation and ribosome translational initiation (Ref. 4 of the main text). The initiation time of various proteins were different (~ 1702s for HrpL_PS_, ~ 1791s for HopG1_PS_, ~ 1699 s for HrpR_PS_, ~ 2454 s for HopX1_PS_, ~ 2485 s for HrpA2_PS_, ~ 759.8 s for PopN_PA_, ~ 17.03 s for Hpa1_XO_ and ~ 2553 s for HrpG_RS_). **Figure S8.** *T*_*test*_*s* of Hpa1_XO_ and HrpG_RS_ in 1× T3SS-repressing conditions were much longer than those in 1× T3SS-inducing conditions. The red line and blue line denoted the time containing both transcription and translation (*T*_*test*_) in 1× T3SS-inducing conditions and 1× T3SS-repressing conditions, respectively. (**A**) *T*_*test*_*s* of *hpa1*_*XO*_-pro-CDS-*lux* reporter in 1× XOM2 and 1× M210 were ~ 3.352 min and ~ 300.4 min. (**B**) *T*_*test*_*s* of *hrpG*_*RS*_-pro-CDS-*lux* reporter in 1× MM_RS_ and 1× B medium were ~ 42.35 s and ~ 181.2 s. **Table S1.** Bacterial strains, plasmid, and primers used in this study.

## Data Availability

All data generated or analysed during this study are included in this published article and its additional files.
