# Supplementary material for: Maintenance of tRNA and elongation factors supports T3SS proteins translational elongations in pathogenic bacteria during nutrient starvation
Source: Cell Biosci. 2022 Sep 5;12:147. doi: 10.1186/s13578-022-00884-6 (PMC9446538; doi:10.1186/s13578-022-00884-6)
Supplement: Supplementary file 1 — Additional file 1: Figure S1. Induction curves of PopNPA14 and non-T3SS proteins (AdhBPS, AlpAPA, MexAPA14, GumBXO and SucCRS) induced in 1× T3SS-inducing conditions and 1× T3SS-repressing conditions. (A) The red curve showed the delayed expression levels of adhBPS-pro-CDS-lux reporter in 1× MM compared with that in 1× KB. (B) The red curve showed the slightly fast expression levels of alpAPA-pro-CDS-lux reporter in LB + 5 mM EGTA compared with that in LB. (C) The red curve showed the delayed expression levels of gumBXO-pro-CDS-lux reporter in 1× XOM2 compared with that in 1× M210. (D) The red curve showed the delayed expression levels of sucCRS-pro-CDS-lux reporter in 1× MMRS compared with that in 1× B. (E) The red curve showed the slightly fast expression levels of popNPA14-pro-CDS-lux reporter in LB + 5 mM EGTA compared with that in LB. (F) The red curve showed the slightly fast expression levels of mexAPA14-pro-CDS-lux reporter in LB + 5 mM EGTA compared with that in LB. (G) The expression of HrpGRS in MMRS at 50 and 60 min were induced rapidly so that the synthesis time of HrpGRS was not calculated. Figure S2. Expression levels of T3SS regulators, effectors and structural protein of P. syringae, X. oryzae and R. solanacearum in 0.5× T3SS-inducing conditions were less stable than that in 1× T3SS-inducing conditions. The red curve and green curve denoted the expression levels in 1× and 0.5× T3SS-inducing conditions, respectively. (A) The expression levels of hrpLPS-pro-CDS-lux reporter were extremely low in 0.5× MM compared with those in 1× MM. (B-D, F) The expression levels of hopG1PS, hrpRPS, hopX1PS and hrpGRS-pro-CDS-lux reporters in 0.5× T3SS-inducing conditions rapidly increased, but declined to a low level immediately compared with those in 1× T3SS-inducing conditions. (E) The expression levels of hpa1XO-pro-CDS-lux reporter in 0.5× XOM2 were slightly lower than those in 1× XOM2. Figure S3. Translational elongation rate of PopNPA14 and non-T3SS proteins (A [file 13578_2022_884_MOESM1_ESM.docx]

Maintenance of tRNA and Elongation Factors Supports T3SS Proteins Translational Elongations in Pathogenic Bacteria during Nutrient Starvation

**Additional file 1**

Yue Sun^1^, Xiaolong Shao^1,2^, Yingchao Zhang^1^, Liangliang Han^1^, Jiadai Huang^1^, Yingpeng Xie^1^, Jingui Liu^1^, Xin Deng^1,3^

^1^ Department of Biomedical Sciences, City University of Hong Kong, Kowloon Tong, Hong Kong SAR, China.

^2^ College of Plant Protection, Laboratory of Plant Immunity, Key Laboratory of

Integrated Management of Crop Diseases and Pests, Nanjing Agricultural University,

No.1 Weigang, Nanjing, Jiangsu 210095, China.

^3^ Shenzhen Research Institute, City University of Hong Kong, Shenzhen, 518057, China.

^*^ To whom correspondence should be addressed to Dr. Xin Deng, Tel: +852 3442 5693; Fax: +852 3442 0549; Email: xindeng@cityu.edu.hk

**
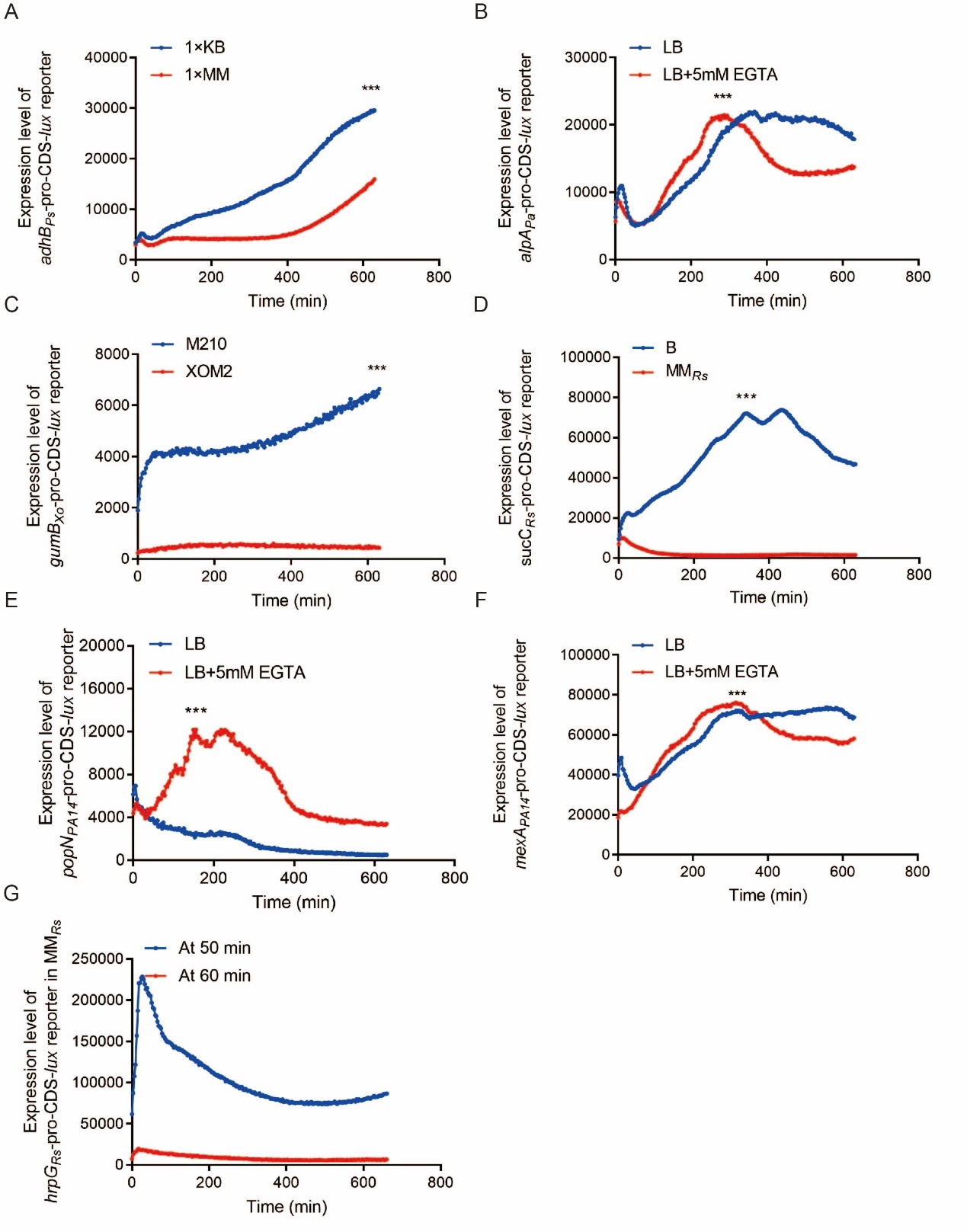
**

**Figure S1. Induction curves of PopN_PA14_ and non-T3SS proteins (AdhB_PS_, AlpA_PA_, MexA_PA14_, GumB_XO_ and SucC_RS_) induced in** **1× T3SS-inducing conditions and 1× T3SS-repressing conditions.** (**A**) The red curve showed the delayed expression levels of *adhB**_PS_*-pro-CDS-*lux* reporter in 1× MM compared with that in 1× KB. (**B**) The red curve showed the slightly fast expression levels of *alpA_PA_*-pro-CDS-*lux* reporter in LB+ 5mM EGTA compared with that in LB. (**C**) The red curve showed the delayed expression levels of *gumB_XO_*-pro-CDS-lux reporter in 1× XOM2 compared with that in 1× M210. **(D)** The red curve showed the delayed expression levels of *sucC_RS_*-pro-CDS-lux reporter in 1× MM_RS_ compared with that in 1× B. **(E)** The red curve showed the slightly fast expression levels of *popN_PA14_*-pro-CDS-*lux* reporter in LB+ 5mM EGTA compared with that in LB. (**F**) The red curve showed the slightly fast expression levels of *mexA_PA14_*-pro-CDS-*lux* reporter in LB+ 5mM EGTA compared with that in LB. (**G**) The expression of HrpG_RS_ in MM_RS_ at 50 and 60min were induced rapidly so that the synthesis time of HrpG_RS_ was not calculated.

**
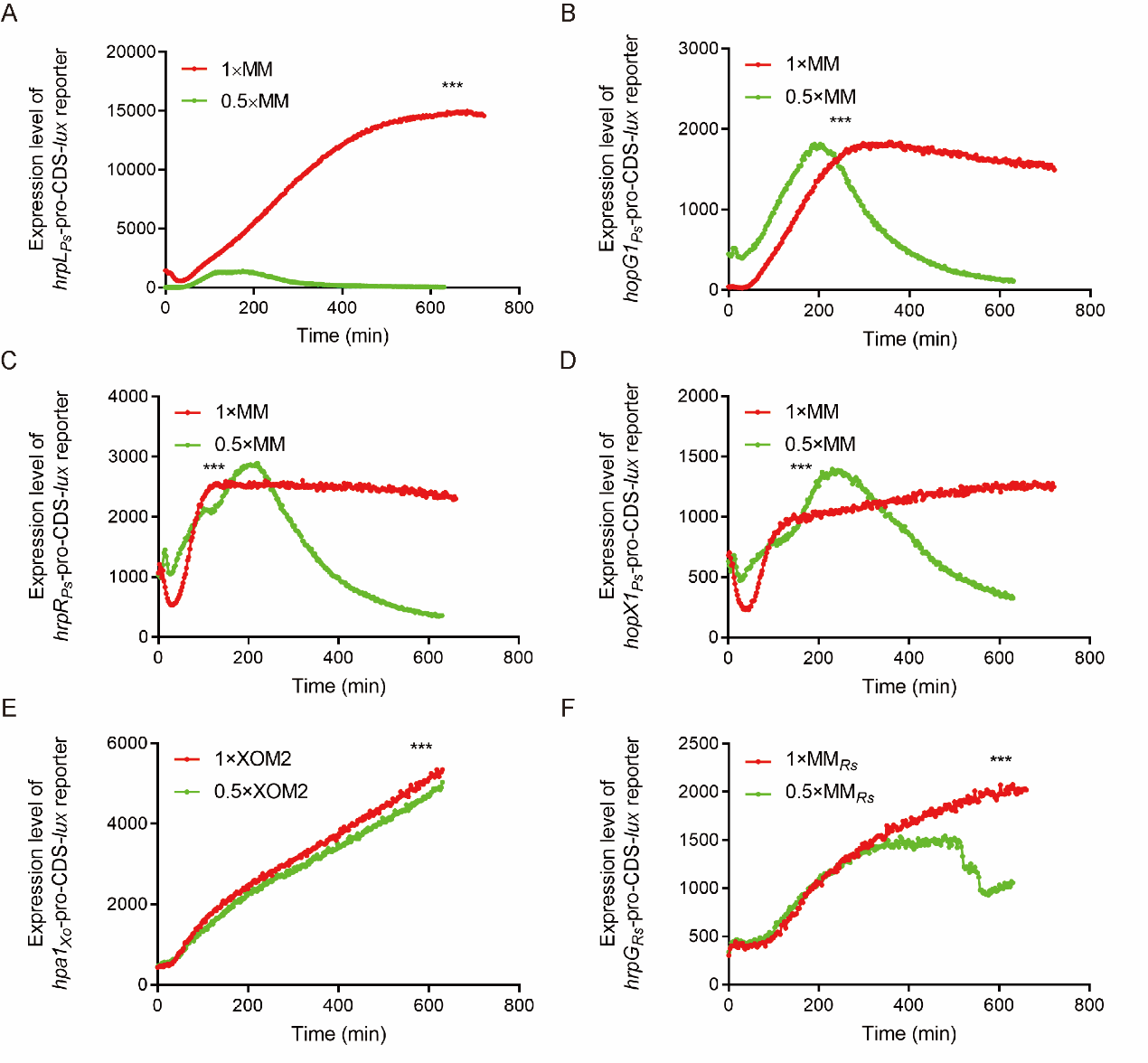
**

**Figure S2. Expression levels of T3SS regulators, effectors and structural protein of *P. syringae*, *X. oryzae* and *R. solanacearum* in 0.5× T3SS-inducing conditions were less stable than that in 1× T3SS-inducing conditions.** The red curve and green curve denoted the expression levels in 1× and 0.5× T3SS-inducing conditions, respectively. (**A**) The expression levels of *hrpL_PS_*-pro-CDS-*lux* reporter were extremely low in 0.5× MM compared with those in 1× MM. (**B-D, F**) The expression levels of *hopG1_PS_*, *hrpR_PS_*, *hopX1_PS_* and *hrpG_RS_*-pro-CDS-*lux* reporters in 0.5× T3SS-inducing conditions rapidly increased, but declined to a low level immediately compared with those in 1× T3SS-inducing conditions. (**E**) The expression levels of *hpa1_XO_*-pro-CDS-*lux* reporter in 0.5× XOM2 were slightly lower than those in 1× XOM2.

**
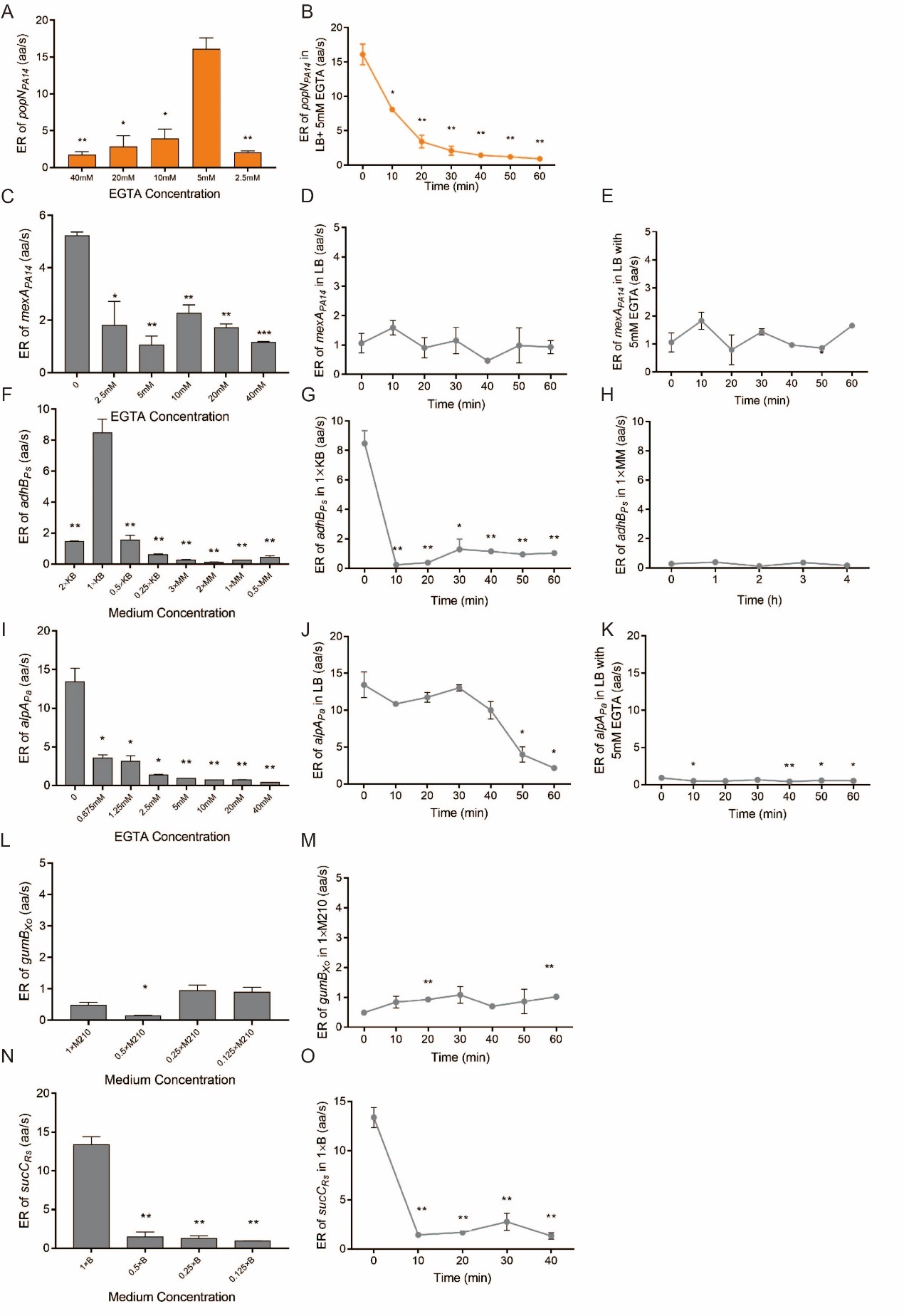
**

**Figure S3. Translational elongation rate of PopN_PA14_ and non-T3SS proteins (AdhB_PS_, AlpA_PA_, MexA_PA14_, GumB_XO_ and SucC_RS_) in multiple nutrition conditions and at various time. (A-B)** Translational ERs of PopN_PA14_ under different EGTA concentrations and at various culture time (0-60 min). (**C**) The translational ERs of MexA_PA14_ in LB with EGTA (~1.0-~2.2 aa/s) were much lower than that in LB (~5.2 aa/s) with no significant difference. (**D-E**) The translational ERs of MexA_PA14_ in both LB and LB with 5mM EGTA during various culture times kept at a low level (~0.5- ~2.0 aa/s and ~0.8- ~1.8 aa/s, respectively). (**F**) The translational ER of AdhB_PS_ in 1× MM (~8 aa/s) was more than 4-fold than those in richer or poorer nutrition conditions (less than 2 aa/s). (**G**) In 1× KB, the translational ER of AdhB_PS_ at the beginning of culture (~8 aa/s) had significant difference than those at various culture time (less than 2 aa/s). (**H**) In 1× MM, the translational ERs of AdhB_PS_ at 0-4 h of culture were much lower than that in 1× KB, which had no difference. (**I**) The translational ER of AlpA_PA_ in LB (~13 aa/s) was more than 3-fold than those in LB+ EGTA (less than 5 aa/s). (**J**) In LB, the translational ERs of AlpA_PA_ at 0-40 min of culture (10-15 aa/s) had no difference. The ER decreased rapidly at 50-60 min (less than 5 aa/s). (**K**) In LB+ 5mM EGTA, the translational ERs of AlpA_PA_ were much lower than those in LB. **(L)** The translational ER of GumB_XO_ in 1× M210 (~0.5 aa/s) had no significant differences with those in 0.5× M210 and 0.125× M210 (~0.8 aa/s). (**M**) The translational ERs of GumB_XO_ at 10, 30, 40 and 50 min (~0.7-0.8 aa/s) showed no differences with that at 0 min (~0.5 aa/s) apart from those at 20 and 60 min (~0.9-1.0 aa/s). (**N**) The ERs of SucC_RS_ in 0.5×, 0.25× and 0.125× B (~0.9-1.4 aa/s) were much lower than that in 1× B (~13.4 aa/s). (**O**) The translational ERs of SucC_RS_ for 10-60 minutes (~1.3-2.7 aa/s) were much lower than that at 0 min (~13.4 aa/s).

**
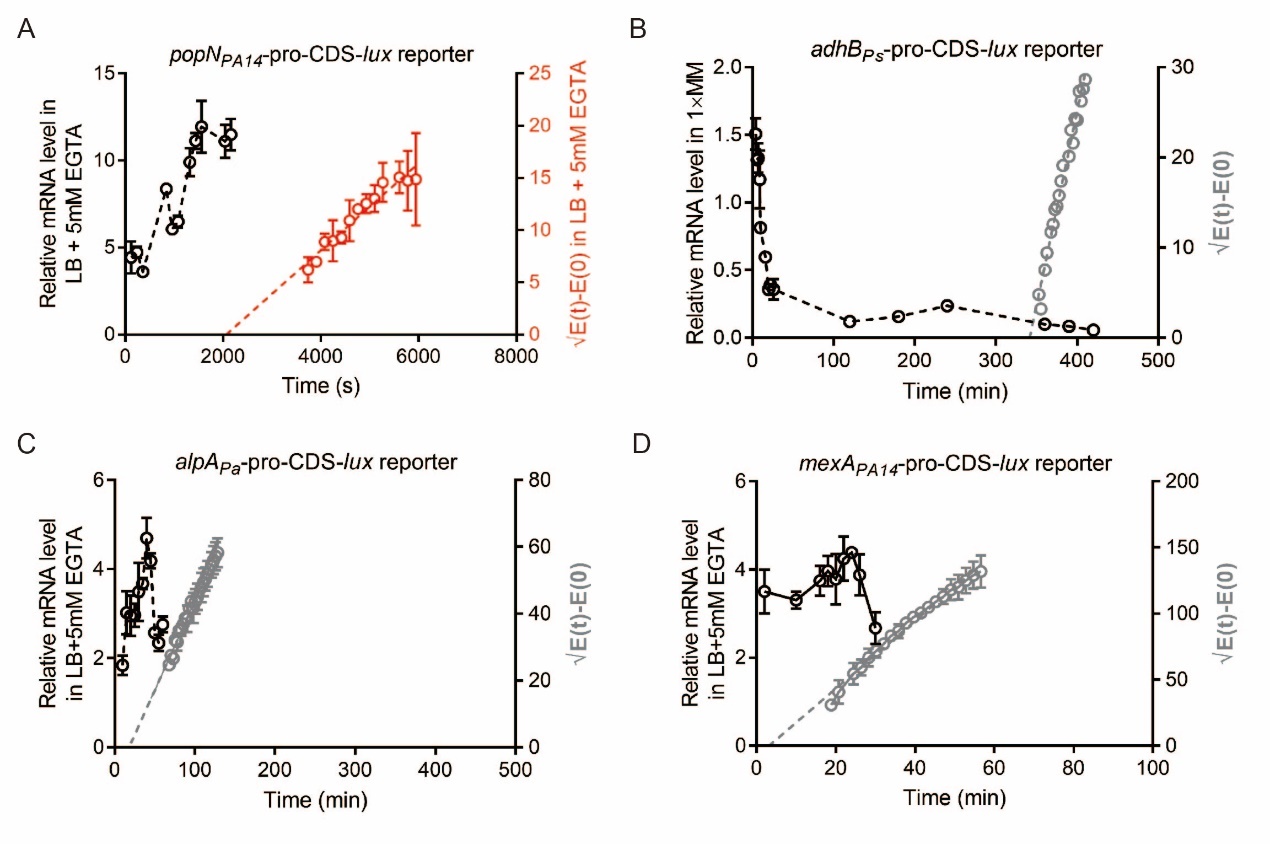
**

**Figure S4. Induction assay of full-length PopN_PA14_ and non-T3SS proteins** **(AdhB_PS_, AlpA_PA_ and MexA_PA14_) mRNAs in 1× T3SS-inducing conditions.** (**A**) The mRNA synthesis level of *popN_PA14_* was rapidly induced in LB with 5mM EGTA. $\sqrt{E\left( t \right)-E(0)}$ plot showed that *T_test_* of PopN_PA14_ was ~2191 s in LB with 5mM EGTA. (**B**) The mRNA synthesis level of *adhB_PS_* rapidly decreased in 1× MM and maintained a low level within 26-420 min. $\sqrt{E\left( t \right)-E(0)}$ plot showed that *T_test_* of AdhB_PS_ was ~343.7 min in 1× MM. (**C**) The mRNA synthesis level of *alpA_PA_* increased after adding EGTA and dropped to the starting level. $\sqrt{E\left( t \right)-E(0)}$ plot showed that *T_test_* of AlpA_PA_ was ~47 s in LB+ 5mM EGTA. (**D**) The mRNA synthesis level of *mexA_PA14_* increased after adding EGTA and dropped to the starting level. $\sqrt{E\left( t \right)-E(0)}$ plot showed that *T_test_* of MexA_PA14_ was ~5 min in LB+ 5mM EGTA.


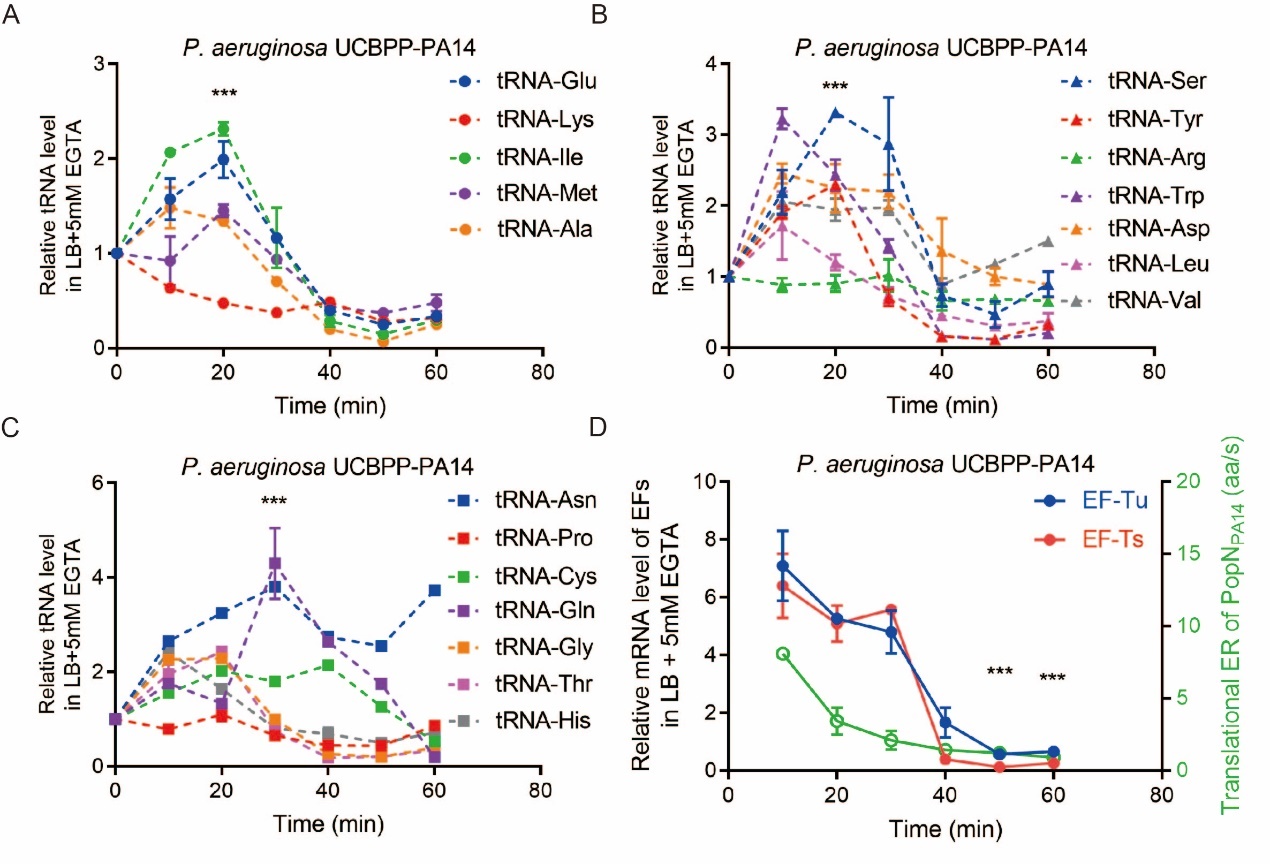


**Figure S5. Relative expression levels of tRNAs and EFs of PA14 in LB with 5mM EGTA.** (**A-C**) tRNAs of PA14 were detected at 10, 20, 30, 40, 50, 60 min. The data at 0 min for each individual tRNA was set as 1. (**D**) The relative EFs levels of PA14 were detected by qRT-PCR. The EFs levels were shown together with the translational ER of PopN_PA14_ in LB with 5mM EGTA. The translational ERs at specific times were shown as green hollow symbols. The standard deviations were shown but were very small in the plot. ∗ p < 0.05; ∗∗ p < 0.01; and ∗∗∗ p < 0.001. Results were indicated in mean ± SD. All experiments were repeated at least three times.


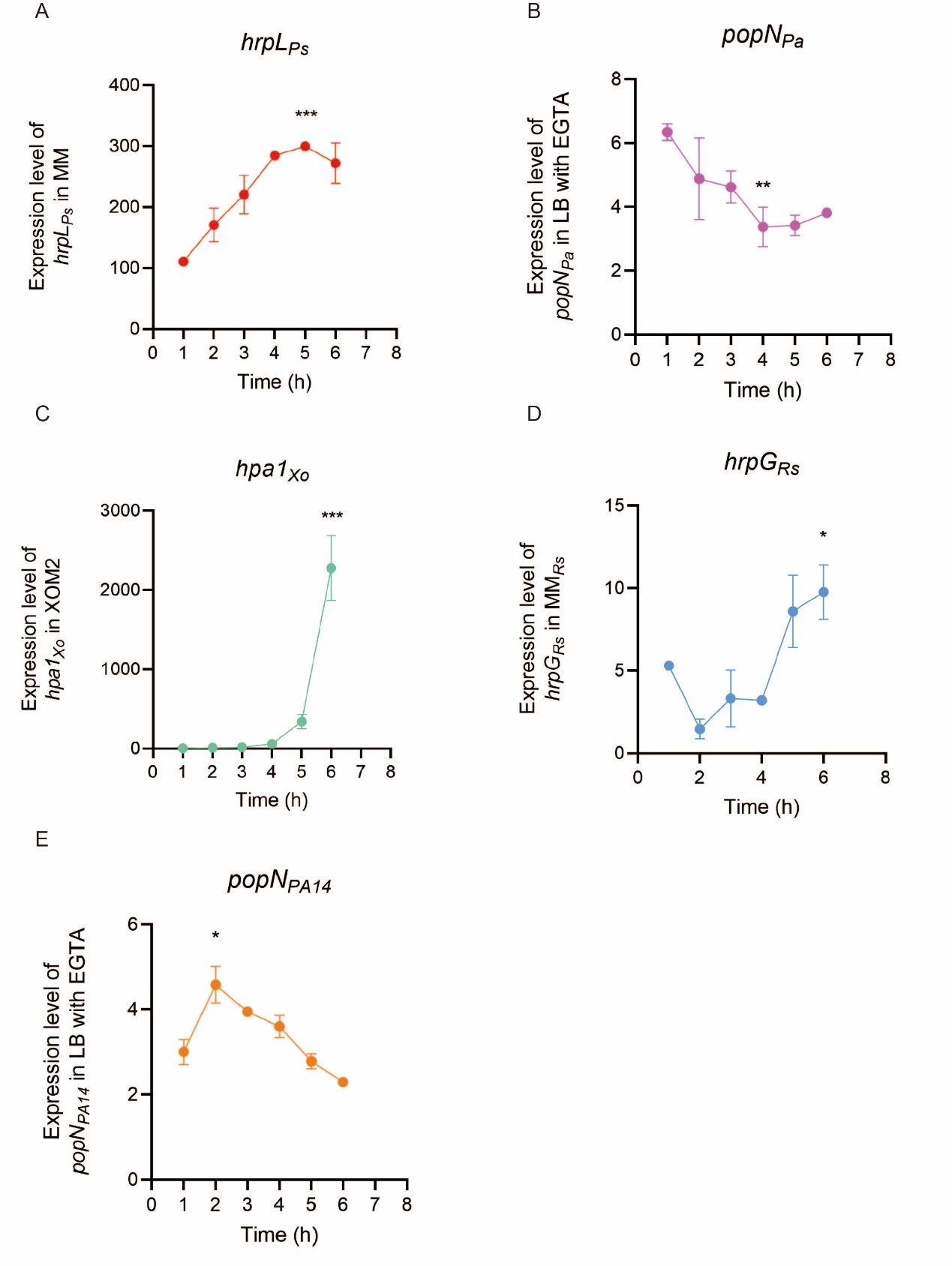


**Figure S6. The expression levels of T3SS genes during 0-6-hours inducing culture.** (**A-E**) The expression levels of *hrpL_PS_*, *popN_PA_*, *hpa1_XO_*, *hrpG_RS_* and *popN_PA14_* in 1× T3SS-inducing conditions for 0-6 hours.

**
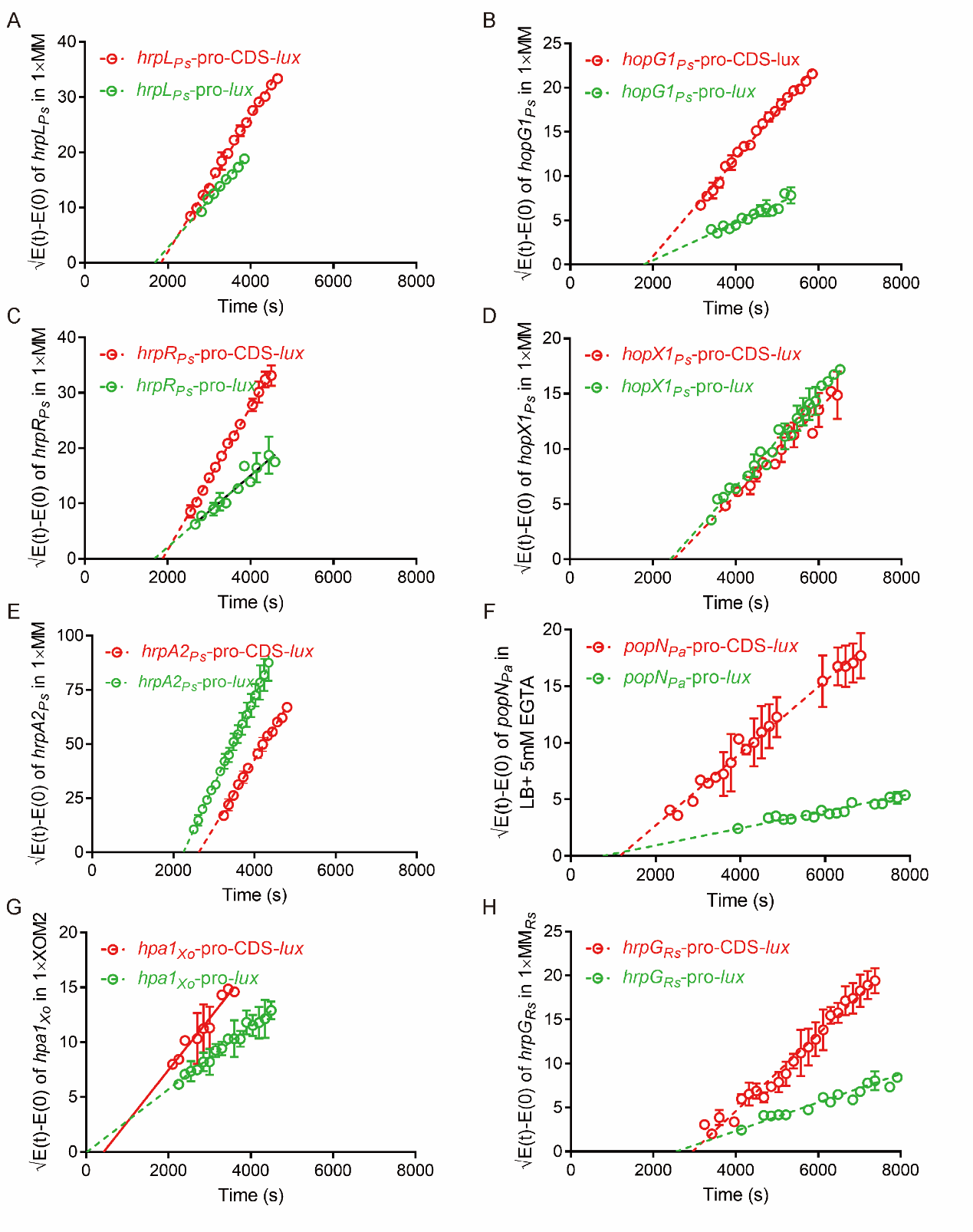
**

**Figure S7.** **The Schleif plot of T3SS regulators, effectors and structural protein of *P. syringae*, *P. aeruginosa*, *X. oryzae* and *R. solanacearum* in 1× T3SS-inducing conditions.** The Schleif plot was used to detect the translational time of the first newly synthesized protein in 1× T3SS-inducing conditions. The square root of newly synthesized protein ($\sqrt{E\left( t \right)-E(0)}$) was linear correlated with time during the initial dozens of minutes. *E(t)* denoted the expression levels of the tested proteins at specific times in 1× T3SS-inducing conditions, and *E(0)* denoted the basal expression levels of the culture. The green line and red line described the linear line of transcriptional fusion reporters and reporters containing both transcription and translation, respectively. The x-intercepts of the green line and red line denoted the time for the initial cost (*T_initiation_*) and the transcription and translation of the tested proteins (*T_test_*), respectively. Therefore, the difference of the two intercepts corresponded to the time to translate the full-length protein molecule (*T_first_*). The initial cost included the sense of cells to nutrient conditions, RNA polymerase transcriptional initiation and ribosome translational initiation (Ref. 4 of the main text). The initiation time of various proteins were different (~1702 s for HrpL_PS_, ~1791 s for HopG1_PS_, ~1699 s for HrpR_PS_, ~2454 s for HopX1_PS_, ~2485 s for HrpA2_PS_, ~759.8 s for PopN_PA_, ~17.03 s for Hpa1_XO_ and ~2553 s for HrpG_RS_).

**
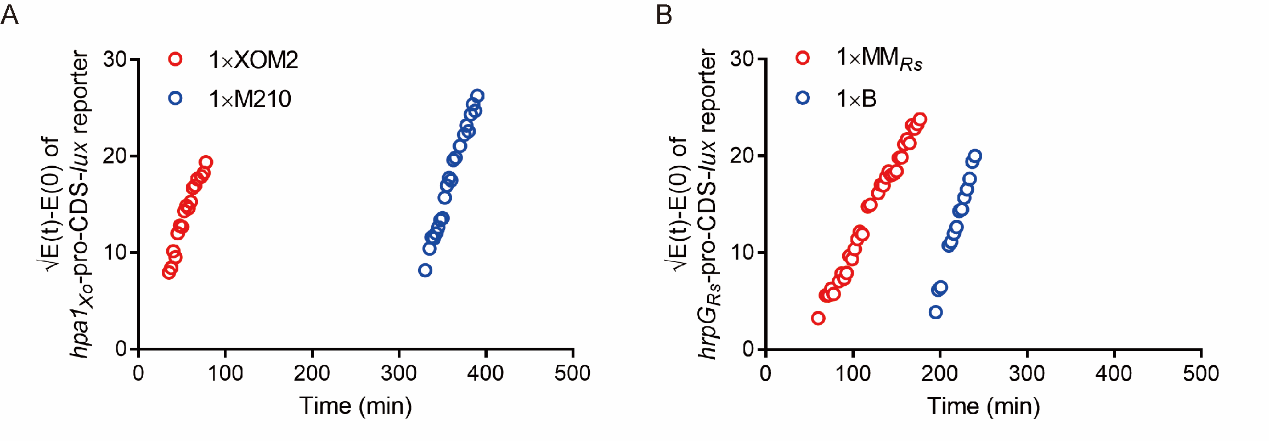
**

**Figure S8. *T_test_s* of Hpa1_XO_ and HrpG_RS_ in 1× T3SS-repressing conditions were much longer than those in** **1× T3SS-inducing conditions.** The red line and blue line denoted the time containing both transcription and translation (*T_test_*) in 1× T3SS-inducing conditions and 1× T3SS-repressing conditions, respectively. (**A**) *T_test_s* of *hpa1_XO_*-pro-CDS-*lux* reporter in 1× XOM2 and 1× M210 were ~3.352 min and ~300.4 min. (**B**) *T_test_s* of *hrpG_RS_*-pro-CDS-*lux* reporter in 1× MM_RS_ and 1× B medium were ~42.35 s and ~181.2 s.

**Table S1. Bacterial strains, plasmid, and primers used in this study.**

| **Description** | **Genotype or relevant phenotype** | **Source or reference** |
| --- | --- | --- |
|  |  |  |
| ***E.coli* DH5α** | supE44 Δlac*U169*(φ80lacZΔM15)*hsdR17recA1 endA1 gyrA96thi-1 relA1*λpir | stratagene |
| ***P. savastanoi pv. phaseolicola* 1448A** | Wild type | Lab stock |
| ***P. aeruginosa* PAO1** | Wild type | Lab stock |
| ***X. oryzae pv. oryzae* PXO99 A** | Wild type | Lab stock |
| ***R. solanacearum* OE1‐1** | Wild type | Lab stock |
|  |  |  |
| **pMS402** | Reporter plasmid carring the promoter less *lux* CDABE | Lab stock |
|  |  |  |
| ***hrpL_Ps_*-pro-CDS-*lux*** | Translational fusion reporter between *hrpL_Ps_* sequence containing promoter and *lux* in pMS402 | This study |
| ***hrpL_Ps_*-pro-*lux*** | Transcriptional fusion reporter between *hrpL_Ps_* promoter and *lux* in pMS402 | This study |
| ***hopG1_Ps_*-pro-CDS-*lux*** | Translational fusion reporter between *hopG1_Ps_* sequence containing promoter and *lux* in pMS402 | This study |
| ***hopG1_Ps_*-pro-*lux*** | Transcriptional fusion reporter between *hopG1_Ps_* promoter and *lux* in pMS402 | This study |
| ***hrpR_Ps_*-pro-CDS-*lux*** | Translational fusion reporter between *hrpR_Ps_* sequence containing promoter and *lux* in pMS402 | This study |
| ***hrpR_Ps_*-pro-*lux*** | Transcriptional fusion reporter between *hrpR_Ps_* promoter and *lux* in pMS402 | This study |
| ***hopX1_Ps_*-pro-CDS-*lux*** | Translational fusion reporter between *hopX1_Ps_* sequence containing promoter and *lux* in pMS402 | This study |
| ***hopX1_Ps_*-pro-*lux*** | Transcriptional fusion reporter between *hopX1_Ps_* promoter and *lux* in pMS402 | This study |
| ***hrpA2_Ps_*-pro-CDS-*lux*** | Translational fusion reporter between *hrpA2_Ps_* sequence containing promoter and *lux* in pMS402 | This study |
| ***hrpA2_Ps_*-pro-*lux*** | Transcriptional fusion reporter between *hrpA2_Ps_* promoter and *lux* in pMS402 | This study |
| ***adhB_Ps_*-pro-CDS-*lux*** | Translational fusion reporter between *adhB_Ps_* sequence containing promoter and *lux* in pMS402 | This study |
| ***adhB_Ps_*-pro-*lux*** | Transcriptional fusion reporter between *adhB_Ps_* promoter and *lux* in pMS402 | This study |
| ***popN_Pa_*-pro-CDS-*lux*** | Translational fusion reporter between *popN_Pa_* sequence containing promoter and *lux* in pMS402 | This study |
| ***popN_Pa_*-pro-*lux*** | Transcriptional fusion reporter between *popN_Pa_* promoter and *lux* in pMS402 | This study |
| ***alpA_Pa_*-pro-CDS-*lux*** | Translational fusion reporter between *alpA_Pa_* sequence containing promoter and *lux* in pMS402 | This study |
| ***alpA_Pa_*-pro-*lux*** | Transcriptional fusion reporter between *alpA_Pa_* promoter and *lux* in pMS402 | This study |
| ***hpa1_Xo_*-pro-CDS-*lux*** | Translational fusion reporter between *hpa1_Xo_* sequence containing promoter and *lux* in pMS402 | This study |
| ***hpa1_Xo_*-pro-*lux*** | Transcriptional fusion reporter between *hpa1_Xo_* promoter and *lux* in pMS402 | This study |
| ***hrpG_Rs_*-pro-CDS-*lux*** | Translational fusion reporter between *hrpG_Rs_* sequence containing promoter and *lux* in pMS402 | This study |
| ***hrpG_Rs_*-pro-*lux*** | Transcriptional fusion reporter between *hrpG_Rs_* promoter and *lux* in pMS402 | This study |
|  |  | This study |
| **Primers** | **Sequence (5’ to 3’)** | This study |
| ***hrpL_Ps_-*pro-CDS-*lux*-5'** | TCGTCTTCACCTCGAGGGGATCCACGGTTCTGAGCCTGGTC | This study |
| ***hrpL_Ps_-*pro-*lux*-3'** | GCGGCCGCAACTAGAGGATCCCGGAAACATGGGCTTACCTT | This study |
| ***hrpL_Ps_-*pro-CDS-*lux*-3'** | GCGGCCGCAACTAGAGGATCCGGCGAACGGGTCAATCTGCT | This study |
| ***hopG1_Ps_*-pro-CDS-*lux*-5'** | TCGTCTTCACCTCGAGGGGATCCTGAACCCAGCCTTTGAAC | This study |
| ***hopG1_Ps_*-pro-*lux*-3'** | GCGGCCGCAACTAGAGGATCCTCTCCTAATGCTATGAGGC | This study |
| ***hopG1_Ps_*-pro-CDS-*lux*-3'** | GCGGCCGCAACTAGAGGATCCCGAATCAGGCTTGTCAACC | This study |
| ***hrpR_Ps_*-pro-CDS-*lux*-5'** | TCGTCTTCACCTCGAGGGGATCCTTCCTTGAAAGGGGACTGC | This study |
| ***hrpR_Ps_*-pro-*lux*-3'** | GCGGCCGCAACTAGAGGATCCCGATCACTCTCACTGTGG | This study |
| ***hrpR_Ps_*-pro-CDS-*lux*-3'** | GCGGCCGCAACTAGAGGATCCTGCAGCAACTCCCAACTC | This study |
| ***hopX1_Ps_*-pro-CDS-*lux*-5'** | TCGTCTTCACCTCGAGGGGATCCATGGGCTTTGCGTCAGTGTC | This study |
| ***hopX1_Ps_*-pro-*lux*-3'** | GCGGCCGCAACTAGAGGATCCATGTTCCTCACTGATTGCGG | This study |
| ***hopX1_Ps_*-pro-CDS-*lux*-3'** | GCGGCCGCAACTAGAGGATCCTCTTCGTGGAGGCATGC | This study |
| ***hrpA2_Ps_*-pro-CDS-*lux*-5'** | TCGTCTTCACCTCGAGGGGATCCGATTTTTTGCAGAGCGCTGG | This study |
| ***hrpA2_Ps_*-pro-*lux*-3'** | GCGGCCGCAACTAGAGGATCCAACGTGATACCCCTTGGTCTG | This study |
| ***hrpA2_Ps_*-pro-CDS-*lux*-3'** | GCGGCCGCAACTAGAGGATCCGAACTGGACGACCGAGTTCC | This study |
| ***adhB_Ps_*-pro-CDS-*lux*-5'** | TCGTCTTCACCTCGAGGGGATCCTGAAACAGCCACCCTGCAC | This study |
| ***adhB_Ps_*-pro-*lux*-3'** | GCGGCCGCAACTAGAGGATCCGACGGACCCTCGTGGGTAGC | This study |
| ***adhB_Ps_*-pro-CDS-*lux*-3'** | GCGGCCGCAACTAGAGGATCCTGAATCTGCTCAAGATCGGC | This study |
| ***popN_Pa_*-pro-CDS-*lux*-5'** | TCGTCTTCACCTCGAGGGGATCCATCGGTTCGGCTGGAGT | This study |
| ***popN_Pa_*-pro-*lux*-3'** | GCGGCCGCAACTAGAGGATCCCTGTGGTTCCTGGTCTGCAA | This study |
| ***popN_Pa_*-pro-CDS-*lux*-3'** | GCGGCCGCAACTAGAGGATCCAAGGCCCGTATGCCATG | This study |
| ***alpA_Pa_*-pro-CDS-*lux*-5'** | TCGTCTTCACCTCGAGGGGATCCAGGTGGAAAGACTAAGGGTG | This study |
| ***alpA_Pa_*-pro-*lux*-3'** | GCGGCCGCAACTAGAGGATCCGGTATTCCCTCCTTGGCCGG | This study |
| ***alpA_Pa_*-pro-CDS-*lux*-3'** | GCGGCCGCAACTAGAGGATCCGCGCTCGCCCACGATCCCCA | This study |
| ***hpa1_Xo_*-pro-CDS-*lux*-5'** | TCGTCTTCACCTCGAGGGGATCCTACCGACATACTGGCATGCG | This study |
| ***hpa1_Xo_*-pro-*lux*-3'** | GCGGCCGCAACTAGAGGATCCATCAACAGCCGTGCCTAGAC | This study |
| ***hpa1_Xo_*-pro-CDS-*lux*-3'** | GCGGCCGCAACTAGAGGATCCTACTGCATCGATGCGCTGTC | This study |
| ***hrpG_Rs_*-pro-CDS-*lux*-5'** | TCGTCTTCACCTCGAGGGGATCCAATCGTTCAAGTGGTGGCCG | This study |
| ***hrpG_Rs_*-pro-*lux*-3'** | GCGGCCGCAACTAGAGGATCCATGCTCCTGAAGCGTCAGTC | This study |
| ***hrpG_Rs_*-pro-CDS-*lux*-3'** | GCGGCCGCAACTAGAGGATCCTCGTTGAACTGCTTGCGG | This study |
|  |  |  |
| **mRNA** | **Sequence (5' to 3')** |  |
| ***hrpL_Ps_*-pro-CDS-*lux-*F** | CTGGGAAGACGACGTTCATT |  |
| ***hrpL_Ps_*-pro-CDS-*lux*-R** | ATCTCCAGCGACACTTCCAG |  |
| ***hopG1_Ps_*-pro-CDS-*lux*-F** | AATCATGCACGCTTGTTGAG |  |
| ***hopG1_Ps_*-pro-CDS-*lux*-R** | TCCCTAAGAGTGTGGCGTTC |  |
| ***hrpR_Ps_*-pro-CDS-*lux*-F** | TGCTTATTCAGGATGCGTTG |  |
| ***hrpR_Ps_*-pro-CDS-*lux*-R** | AACTCCCAACTCCTTCATGC |  |
| ***hopX1_Ps_*-pro-CDS-*lux*-F** | TGGCGAACATGCTCGTATAG |  |
| ***hopX1_Ps_*-pro-CDS-*lux*-R** | TGGCGAACATGCTCGTATAG |  |
| ***hrpA2_Ps_*-pro-CDS-*lux*-F** | TGCGAACGAAGAGAACGGC |  |
| ***hrpA2_Ps_*-pro-CDS-*lux*-R** | GAACTGGACGACCGAGTTCC |  |
| ***adhB_Pa_*-pro-CDS-*lux*-F** | TGGGCTGCAATATTCGTGGT |  |
| ***adhB_Pa_*-pro-CDS-*lux*-R** | ATCTGCTCAAGATCGGCACG |  |
| ***popN_Pa_*-pro-CDS-*lux*-F** | TTCCTGCAGAAAGCCCTGAG |  |
| ***popN_Pa_*-pro-CDS-*lux*-R** | AAGGCCCGTATGCCATG |  |
| ***alpA_Pa_*-pro-CDS-*lux*-F** | TTGCTGATGCGCATGTTCCG |  |
| ***alpA_Pa_*-pro-CDS-*lux*-R** | ATCGGCTCCTCCAGCCGCTG |  |
| ***hpa1_Xo_*-pro-CDS-*lux*-F** | ATTCACCCAGATGCTGATGC |  |
| ***hpa1_Xo_*-pro-CDS-*lux*-R** | TACTGCATCGATGCGCTGTC |  |
| ***hrpG_Rs_*-pro-CDS-*lux*-F** | ATATCAACGTGACCGAGCGC |  |
| ***hrpG_Rs_*-pro-CDS-*lux*-F** | TCGTTGAACTGCTTGCGG |  |
|  |  |  |
| **tRNA** | **Sequence (5' to 3')** |  |
| **16s for *Ps*-F** | ATGGTCGGTACAGAGGGTTG |  |
| **16s for *Ps*-R** | TCGCGATTACTAGCGATTCC |  |
| ***Ps*-tRNA-Met-RT-F** | CGCGGGATGGAGCAGTC |  |
| ***Ps*-tRNA-Met-RT-R** | GATTTGAACCGACGACCTTC |  |
| ***Ps*-tRNA-Glu-RT-F** | GTCCCCTTCGTCTAGTGG |  |
| ***Ps*-tRNA-Glu-RT-R** | TGGCGTCCCCTAGGGGACTCGAA |  |
| ***Ps*-tRNA-Lys-RT-F** | GGGTCGTTAGCTCAGTTGGTA |  |
| ***Ps*-tRNA-Lys-RT-R** | TGGTGGGTCGTGTGGGATTCG |  |
| ***Ps*-tRNA-Ile-RT-F** | GGGTCTGTAGCTCAGTTGGTT |  |
| ***Ps*-tRNA-Ile-RT-R** | GGTGGGTCTGGGCAGATTC |  |
| ***Ps*-tRNA-Phe-RT-F** | GCCCAGATAGCTCAGTCGGTA |  |
| ***Ps*-tRNA-Phe-RT-R** | TGGTGCCCAGAGACGGAAT |  |
| ***Ps*-tRNA-Ala-RT-F** | GGGGCTTTAGCTCAGCTGGGA |  |
| ***Ps*-tRNA-Ala-RT-R** | TGGTGGAGCTTAGCGGGAT |  |
| ***Ps*-tRNA-Ser-RT-F** | GGTGAGGTGTCCGAGTGGCTT |  |
| ***Ps*-tRNA-Ser-RT-R** | TGGCGGTGAAGGAGGGATTCG |  |
| ***Ps*-tRNA-Tyr-RT-F** | GGAGGGGTTCCCGAGCGGCCA |  |
| ***Ps*-tRNA-Tyr-RT-R** | TGGTGGAGGGGGAAGGATTC |  |
| ***Ps*-tRNA-Arg-RT-F** | GTGCCCGTAGCTCAGCTGGA |  |
| ***Ps*-tRNA-Arg-RT-R** | TGGCGCACCCGGCAGGACTCGAA |  |
| ***Ps*-tRNA-Trp-RT-F** | AGGTCAGTAGCTCAATTGGCA |  |
| ***Ps*-tRNA-Trp-RT-R** | TGGCAGGTCAGGAGGGAAT |  |
| ***Ps*-tRNA-Asp-RT-F** | GCAGCGGTAGTTCAGTCGG |  |
| ***Ps*-tRNA-Asp-RT-R** | TGGCGCAGCGGACGGGACT |  |
| ***Ps*-tRNA-Leu-RT-F** | GCGGATGTGGTGGAATTGGT |  |
| ***Ps*-tRNA-Leu-RT-R** | TGGTGCGGACGGAGAGACTC |  |
| ***Ps*-tRNA-Val-RT-F** | GGGTGATTAGCTCAGCTGGGAG |  |
| ***Ps*-tRNA-Val-RT-R** | TGGTGGGTGATGACGGGAT |  |
| ***Ps*-tRNA-Asn-RT-F** | TCCGCGATAGCTCAGTTGGT |  |
| ***Ps*-tRNA-Asn-RT-R** | TGGCTCCACGACCTGGACTC |  |
| ***Ps*-tRNA-Pro-RT-F** | CGGGGTATAGCGCAGTCCGGT |  |
| ***Ps*-tRNA-Pro-RT-R** | TGGTCGGGGTAAGGGGATTC |  |
| ***Ps*-tRNA-Cys-RT-F** | GGCCGAGTAGCAAAATGGTT |  |
| ***Ps*-tRNA-Cys-RT-R** | TGGAGGCCGAGGTCGGAATC |  |
| ***Ps*-tRNA-Gln-RT-F** | AGGGGCGTCGCCAAGCGGTAAG |  |
| ***Ps*-tRNA-Gln-RT-R** | TGGCAGGGGCGGCTGGATTC |  |
| ***Ps*-tRNA-Thr-RT-F** | GCTCTTGTAGCTCAGTTGGTA |  |
| ***Ps*-tRNA-Thr-RT-R** | TGGAGCTCTTGAGCGGATT |  |
| ***Ps*-tRNA-Gly-RT-F** | GCGGGAATAGCTCAGTTGGTAG |  |
| ***Ps*-tRNA-Gly-RT-R** | TGGAGCGGGAAACGAGACTC |  |
| ***Ps*-tRNA-His-RT-F** | TGGGCGTAGCTCAGTTGGTA |  |
| ***Ps*-tRNA-His-RT-R** | ACGACCGCAGGAGTCACA |  |
|  |  |  |
| **16s for *Pa*-F** | GACACGGTCCAGACTCCTAC |  |
| **16s for *Pa*-R** | AACTTACTGCCCTTCCTCCC |  |
| ***Pa*-tRNA-Met-RT-F** | GGCCTATAGCTCAGTCGGTTA |  |
| ***Pa*-tRNA-Met-RT-R** | CCGTGGACCAAAGGATTATG |  |
| ***Pa*-tRNA-Glu-RT-F** | CCCCTTCGTCTAGTGGCCTA |  |
| ***Pa*-tRNA-Glu-RT-R** | GGACTCGAACCCCTGTTACC |  |
| ***Pa*-tRNA-Lys-RT-F** | TCGGTAGAGCAGTTGGCTTT |  |
| ***Pa*-tRNA-Lys-RT-R** | TGGTGGGTCGTGTAGGATTC |  |
| ***Pa*-tRNA-Ile-RT-F** | GTTAGAGCGCACCCCTGATA |  |
| ***Pa*-tRNA-Ile-RT-R** | GGTGGGTCTGGGCAGATT |  |
| ***Pa*-tRNA-Phe-RT-F** | TGCCCAGGTAGCTCAGTTG |  |
| ***Pa*-tRNA-Phe-RT-R** | CGACACGGGGATTTTCAAT |  |
| ***Pa*-tRNA-Ala-RT-F** | CCATAGCTCAGCTGGGAGAG |  |
| ***Pa*-tRNA-Ala-RT-R** | GAGCCAAGGAGGATCGAACT |  |
| ***Pa*-tRNA-Ser-RT-F** | GTGTGGCCGAGTGGTTTAAG |  |
| ***Pa*-tRNA-Ser-RT-R** | GGAAGAGTCTCCCCTTCGAC |  |
| ***Pa*-tRNA-Tyr-RT-F** | GGCCAAAGGGATCAGACTGT |  |
| ***Pa*-tRNA-Tyr-RT-R** | CTGGTGGAGGGAGAAGGATT |  |
| ***Pa*-tRNA-Arg-RT-F** | GTTCGATAGCTCAGCCTGGT |  |
| ***Pa*-tRNA-Arg-RT-R** | CGCGACAGACCATTTCGT |  |
| ***Pa*-tRNA-Trp-RT-F** | CCAGTAGCTCAATTGGCAGA |  |
| ***Pa*-tRNA-Trp-RT-R** | GAATCGAACCCCCAACCT |  |
| ***Pa*-tRNA-Asp-RT-F** | CAGCGGTAGTTCAGTCGGTTA |  |
| ***Pa*-tRNA-Asp-RT-R** | GCGTGACAGGCCGGTATT |  |
| ***Pa*-tRNA-Leu-RT-F** | TGGTGGAATTGGTAGACACG |  |
| ***Pa*-tRNA-Leu-RT-R** | ATGGCCACTACCACCTCAAG |  |
| ***Pa*-tRNA-Val-RT-F** | TAGCTCAGCTGGGAGAGCAT |  |
| ***Pa*-tRNA-Val-RT-R** | GCCGACCCTCTGCTTGTAA |  |
| ***Pa*-tRNA-Asn-RT-F** | GCTCAGTCGGTAGAGCAAATG |  |
| ***Pa*-tRNA-Asn-RT-R** | CCAGGGACCCAATGATTAAC |  |
| ***Pa*-tRNA-Pro-RT-F** | CTTGGTAGCGCGTCTCGT |  |
| ***Pa*-tRNA-Pro-RT-R** | ATTCGAACCAGCGACCTTCT |  |
| ***Pa*-tRNA-Cys-RT-F** | GGATTGCAAATCCGTGAAC |  |
| ***Pa*-tRNA-Cys-RT-R** | GCTGAGGTCGGAATCGAAC |  |
| ***Pa*-tRNA-Gln-RT-F** | GGTAAGGCAGCAGGTTTTGA |  |
| ***Pa*-tRNA-Gln-RT-R** | ATTCGAACCAACGCATGG |  |
| ***Pa*-tRNA-Gly-RT-F** | TGGTAGAACCTCAGCCTTCC |  |
| ***Pa*-tRNA-Gly-RT-R** | GAATCGAACCCGCATCATC |  |
| ***Pa*-tRNA-Thr-RT-F** | TGGTAGAGCACACCCTTGGT |  |
| ***Pa*-tRNA-Thr-RT-R** | ATTTGAACCGCTGACCTCAC |  |
| ***Pa*-tRNA-His-RT-F** | GGGCGTAGCTCAGTTGGTAG |  |
| ***Pa*-tRNA-His-RT-R** | ACACCAGGAGCCACAATCCT |  |
|  |  |  |
| ***Xoo*-gyrB-RT-F** | CTGACCGCATTGATCGACCT |  |
| ***Xoo*-gyrB-RT-R** | TGATCGGGTGGCTCTTGTTG |  |
| ***Xoo*-tRNA-Met-RT-F** | CTCAGCCGGTTAGAGCACA |  |
| ***Xoo*-tRNA-Met-RT-R** | CGACCCCAGCATTATGAGTG |  |
| ***Xoo*-tRNA-Glu-RT-F** | TCCCTTCGTCTAATGGTTAGGA |  |
| ***Xoo*-tRNA-Glu-RT-R** | CCCCTGTTTTAGCCTTGAGAG |  |
| ***Xoo*-tRNA-Lys-RT-F** | GCTCAGTCGGTAGAGCAGAAG |  |
| ***Xoo*-tRNA-Lys-RT-R** | CGAACCATCGACCAAAAGAT |  |
| ***Xoo*-tRNA-Ile-RT-F** | ACCCCTGATAAGGGTGAGGT |  |
| ***Xoo*-tRNA-Ile-RT-R** | TGGGTCTGGGTAGACTCGAA |  |
| ***Xoo*-tRNA-Phe-RT-F** | GTTGGTAGAGCAGGGGATTG |  |
| ***Xoo*-tRNA-Phe-RT-R** | CCGAGGACGGAATCGAAC |  |
| ***Xoo*-tRNA-Ala-RT-F** | GGTAGCTCAGCTGGGAGAG |  |
| ***Xoo*-tRNA-Ala-RT-R** | GATCGAACCCTCGACCTTC |  |
| ***Xoo*-tRNA-Ser-RT-F** | CCCCTGCTAAGGGAGTATAGG |  |
| ***Xoo*-tRNA-Ser-RT-R** | GGGATTCGAACCCTCGATAG |  |
| ***Xoo*-tRNA-Tyr-RT-F** | AACGGGGGCAGACTGTAAAT |  |
| ***Xoo*-tRNA-Tyr-RT-R** | GAACCACCGAAGGCGTAA |  |
| ***Xoo*-tRNA-Arg-RT-F** | CCGTAGCTCAGCTGGATAGAGT |  |
| ***Xoo*-tRNA-Arg-RT-R** | GACCGCCTGGTTCGTA |  |
| ***Xoo*-tRNA-Trp-RT-F** | TAGCTCAATTGGCAGAGCAG |  |
| ***Xoo*-tRNA-Trp-RT-R** | AACCTGCGGTTTTGGAGAC |  |
| ***Xoo*-tRNA-Asp-RT-F** | GGAGCGGTAGTTCAGCTGGT |  |
| ***Xoo*-tRNA-Asp-RT-R** | CGTGACAGGCCAGCATTC |  |
| ***Xoo*-tRNA-Leu-RT-F** | ACGCACTAGCTTCAGGTGCT |  |
| ***Xoo*-tRNA-Leu-RT-R** | GACTCGAACCTCCACGAAGT |  |
| ***Xoo*-tRNA-Val-RT-F** | CGGTTAGCTCAGCGGTAGA |  |
| ***Xoo*-tRNA-Val-RT-R** | TGGGCGGTACAGGGTT |  |
| ***Xoo*-tRNA-Asn-RT-F** | CTCAGCCGGTTAGAGCACTT |  |
| ***Xoo*-tRNA-Asn-RT-R** | GTTGGACTCGAACCAACGAC |  |
| ***Xoo*-tRNA-Pro-RT-F** | GGGGTGTAGCTCAGTCTGGT |  |
| ***Xoo*-tRNA-Pro-RT-R** | GGATTCGAACCTACGACCTC |  |
| ***Xoo*-tRNA-Cys-RT-F** | GGCCTGGTGGTAGAGTGGTTA |  |
| ***Xoo*-tRNA-Cys-RT-R** | CGGAATTGAACCGGCGTA |  |
| ***Xoo*-tRNA-Gln-RT-F** | CCAAGGCACCAGGTTTTG |  |
| ***Xoo*-tRNA-Gln-RT-R** | GCAGGGGCGGTAGGATTC |  |
| ***Xoo*-tRNA-Gly-RT-F** | GCGGGAGTAGTTCAACGGTA |  |
| ***Xoo*-tRNA-Gly-RT-R** | GGGAGACGGGAATCGAAC |  |
| ***Xoo*-tRNA-Thr-RT-F** | GCTCAGTCGGTAGAGCACCT |  |
| ***Xoo*-tRNA-Thr-RT-R** | GCTCACGAAAGGAATCGAAC |  |
| ***Xoo*-tRNA-His-RT-F** | GTGGCTGTAGCTCAGCTGGT |  |
| ***Xoo*-tRNA-His-RT-R** | CCCGACATCTGGAATCACAA |  |
|  |  |  |
| ***Rs*-serC-RT-F** | CCCACCTACGCCATCTATGT |  |
| ***Rs*-serC-RT-R** | GGATGAATGTCGTTGCGATA |  |
| ***Rs*-tRNA-Met-RT-F** | AGCAGTCTGGCAGCTCGT |  |
| ***Rs*-tRNA-Met-RT-R** | GATTTGAACCTGCGACCTTC |  |
| ***Rs*-tRNA-Glu-RT-F** | GGCCTAGGACATCACCCTTT |  |
| ***Rs*-tRNA-Glu-RT-R** | TTCGAACCCCTGTACTCACC |  |
| ***Rs*-tRNA-Lys-RT-F** | GGTCGTTAGCTCAGTCGGTA |  |
| ***Rs*-tRNA-Lys-RT-R** | GCGACCAACGGATTAAAAGT |  |
| ***Rs*-tRNA-Ile-RT-F** | TGGTTAGAGCACCGTCTTGA |  |
| ***Rs*-tRNA-Ile-RT-R** | TGGTGGGTCTGGTAGGACTT |  |
| ***Rs*-tRNA-Phe-RT-F** | CGGTAGCTCAGTTGGTAGAGC |  |
| ***Rs*-tRNA-Phe-RT-R** | GGAATCGAACCACCGACAC |  |
| ***Rs*-tRNA-Ala-RT-F** | GCTATAGCTCAGCTGGGAGAG |  |
| ***Rs*-tRNA-Ala-RT-R** | ATCGAACCGCTGACCTCTT |  |
| ***Rs*-tRNA-Ser-RT-F** | GAAGGCACTCCCCTGCTAA |  |
| ***Rs*-tRNA-Ser-RT-R** | ACCCTCGATCCAGGTTTTG |  |
| ***Rs*-tRNA-Tyr-RT-F** | GCTAAAGGGGGCAGACTGTA |  |
| ***Rs*-tRNA-Tyr-RT-R** | TGGTGGAGAGGGTTGGATT |  |
| ***Rs*-tRNA-Arg-RT-F** | CGCCGTAGCACAATGGATAG |  |
| ***Rs*-tRNA-Arg-RT-R** | CGACAGGAATCGAACCTGTATC |  |
| ***Rs*-tRNA-Trp-RT-F** | GGGTATAGCTCAACTGGCAGA |  |
| ***Rs*-tRNA-Trp-RT-R** | AATCGAACCCCCAACCTTC |  |
| ***Rs*-tRNA-Asp-RT-F** | TAGAATACCGGCCTGTCACG |  |
| ***Rs*-tRNA-Asp-RT-R** | GAGTGGACGGGACTCGAA |  |
| ***Rs*-tRNA-Leu-RT-F** | GCCCGAGTGGTGAAATTG |  |
| ***Rs*-tRNA-Leu-RT-R** | GGCGCAGCGGATTTTAAGT |  |
| ***Rs*-tRNA-Val-RT-F** | TAGAGCACTGCCTTCACACG |  |
| ***Rs*-tRNA-Val-RT-R** | GTGGGTGGTACAGGGATTGA |  |
| ***Rs*-tRNA-Asn-RT-F** | CCCGATAGCTCAGTCGGTAG |  |
| ***Rs*-tRNA-Asn-RT-R** | CAGGGACCTACGGATTAACA |  |
| ***Rs*-tRNA-Pro-RT-F** | GTAGCGCAGCCTGGTAGC |  |
| ***Rs*-tRNA-Pro-RT-R** | ATTTGAACCTCCGACCACCT |  |
| ***Rs*-tRNA-Cys-RT-F** | TGGCAGAGTGGTCATGCAG |  |
| ***Rs*-tRNA-Cys-RT-R** | GAATCGAACCGGCGTACA |  |
| ***Rs*-tRNA-Gln-RT-F** | GTTAAGGCACCGGATTTTGA |  |
| ***Rs*-tRNA-Gln-RT-R** | AGGGGAGGAAGGACTCGAA |  |
| ***Rs*-tRNA-Gly-RT-F** | GCGGGAATAGCTCAGTTGGT |  |
| ***Rs*-tRNA-Gly-RT-R** | AACTCGCGACCTCAACCTT |  |
| ***Rs*-tRNA-Thr-RT-F** | AGTTGGTAGAGCAGCGCATT |  |
| ***Rs*-tRNA-Thr-RT-R** | GCCGGAGATAGGAGTCGAA |  |
| ***Rs*-tRNA-His-RT-F** | GTGGCTGTAGCTCAGTTGGT |  |
| ***Rs*-tRNA-His-RT-R** | CACGACGACCAGAATCACAA |  |
|  |  |  |
| **EF** | **Sequence (5' to 3')** |  |
| ***Ps*-Tuf-RT-F** | ACTGGAAATCGTTGGTCTGC |  |
| ***Ps*-Tuf-RT-R** | TTGAAGAACGGAGTGTGACG |  |
| ***Ps*-Tsf-RT-F** | AACGTCGAGCTGGCTAAAGA |  |
| ***Ps*-Tsf-RT-R** | TTGACTTCCGGGTTCTTGAC |  |
| ***Pa*-TufA-RT-F** | CAAGGAAGAAGGTGGTCGTC |  |
| ***Pa*-TufA-RT-R** | ACTCGATGATCTTGGCAACC |  |
| ***Pa*-Tsf-RT-F** | AGCAGCCTTTCGTGAAGAAC |  |
| ***Pa*-Tsf-RT-R** | GCTACTTGAGCGGCAACTTC |  |
| ***Xoo*-Tuf-RT-F** | AGCCGAAGTCTACGTGCTGT |  |
| ***Xoo*-Tuf-RT-R** | CAACGTCACCGTCATCTTGA |  |
| ***Xoo*-Tsf-RT-F** | CTGAGAAGGACAAGGCCAAG |  |
| ***Xoo*-Tsf-RT-R** | CTTCCACCACCTTTTCGATG |  |
| ***Rs*-Tuf-RT-F** | GACAACGTCGGTATCCTGCT |  |
| ***Rs*-Tuf-RT-R** | GTTGTTGAAGAACGGGGTGT |  |
| ***Rs*-Tsf-RT-F** | GAGATCGTTGCCAAGATGGT |  |
| ***Rs*-Tsf-RT-R** | CGAAGTCGTCCTGCTTTTTC |  |
